# Different Strategies for the Preparation of Galactose-Functionalized Thermo-Responsive Nanogels with Potential as Smart Drug Delivery Systems

**DOI:** 10.3390/polym12092150

**Published:** 2020-09-21

**Authors:** Mirian A. González-Ayón, Angel Licea-Claverie, J. Adriana Sañudo-Barajas

**Affiliations:** 1 Centro de Graduados e Investigación en Química, Tecnológico Nacional de México/Instituto Tecnológico de Tijuana, Apartado Postal 1166, Tijuana 22454, Mexico; mirian.gonzalez@tectijuana.edu.mx; 2 Centro de Investigación en Alimentación y Desarrollo, A. C. Carretera a El dorado Km 5.5, Culiacán 80110, Mexico; adriana@ciad.mx

**Keywords:** thermoresponsive nanogels, RAFT, SFEP, *N*-vinylcaprolactam, galactose-functionalized nanogels

## Abstract

Different synthetic strategies were tested for the incorporation of galactose molecules on thermoresponsive nanogels owing to their affinity for receptors expressed in cancer cells. Three families of galactose-functionalized poly(*N*-vinylcaprolactam) nanogels were prepared with the aim to control the introduction of galactose-moieties into the core, the core-shell interface and the shell. First and second of the above mentioned, were prepared via surfactant free emulsion polymerization (SFEP) by a free-radical mechanism and the third one, via SFEP/reversible addition-fragmentation chain transfer (RAFT) polymerization. Synthetic recipes for the SFEP/free radical method included besides *N*-vinylcaprolactam (NVCL), a shell forming poly(ethylene glycol) methyl ether methacrylate (PEGMA), while the galactose (GAL) moiety was introduced via 6-O-acryloyl-1,2,:3,4-bis-O-(1-methyl-ethylidene)-α-_D_-galactopiranose (6-ABG, protected GAL-monomer): nanogels I, or 2-lactobionamidoethyl methacrylate (LAMA, GAL-monomer): nanogels II. For the SFEP/RAFT methodology poly(2-lactobionamidoethyl methacrylate) as GAL macro-chain transfer agent (PLAMA macro-CTA) was first prepared and on a following stage, the macro-CTA was copolymerized with PEGMA and NVCL, nanogels III. The crosslinker ethylene glycol dimethacrylate (EGDMA) was added in both methodologies for the polymer network construction. Nanogel’s sizes obtained resulted between 90 and 370 nm. With higher content of PLAMA macro-CTA or GAL monomer in nanogels, a higher the phase-transition temperature (T_VPT_) was observed with values ranging from 28 to 46 °C. The ρ-parameter, calculated by the ratio of gyration and hydrodynamic radii from static (SLS) and dynamic (DLS) light scattering measurements, and transmission electron microscopy (TEM) micrographs suggest that core-shell nanogels of flexible chains were obtained; in either spherical (nanogels II and III) or hyperbranched (nanogels I) form.

## 1. Introduction

Carbohydrates play a premier role in a myriad of biological processes [1,2]. With the increasing understanding of carbohydrates roles in biological systems, synthetic carbohydrate-based materials, particularly, glycopolymers, and glyco-nanoparticles, are now receiving enormous attention and have been the subject of intense research in the last few decades. There is a strong interest in the preparation of synthetic glycopolymers of well-defined structures, like nanogels and a wide range of other architectures, with compositions that can mimic biological functions, in a less complex environment [3]. Glycopolymers are synthetic polymers featuring pendant carbohydrate moieties. On the one hand, glycopolymers, owing to their biocompatibility and multivalence, play a vital role in many biological recognition processes such as: cell-cell adhesion [4,5], development of new tissues, and infectious behavior of virus and bacteria [6,7]. On the other hand, nanogels, specifically those sensitive to external stimuli such as temperature and pH, are materials with a wide range of applications mainly in the biomedical area due to their excellent properties, such as biocompatibility and stability in aqueous mediums, for instance, for controlled release of drugs [8]. Moreover, due to their hydrophilic character, they are inert materials resulting in cells and proteins not tending to adhere to their surface. Additionally, their characteristic swelling in aqueous mediums, gives them the ability to absorb, retain, and release, under controlled conditions, some organic molecules (drugs) [9]. Therefore, with the advent of nanotechnology, nanoparticles containing sugar residues on the surface, also received a lot of attention, ranging from studies of carbohydrate/protein interactions [10,11], carbohydrate/carbohydrate interactions [12,13,14,15,16], in vivo cell imaging [17,18], vaccine development, targeted drug delivery, and bioassays [19,20].

Several polymerization methods are available to prepare well-defined glycopolymers. Versatile reversible deactivation radical polymerization techniques such as atom-transfer radical polymerization (ATRP) [21] and reversible addition-fragmentation chain transfer (RAFT) [22,23,24] polymerization have been widely used for the engineering of complex polymeric architectures to synthesize well-defined glycopolymers. RAFT has attracted the attention of many polymer researchers as it allows the synthesis of biocompatible polymers with low dispersity index (Đ), high functionality and more important, the possibility to use unprotected glycomonomers. Different types of carbohydrates such as mannose [25], galactose [26], lactose [27], glucose [28] and dextrin [29] have been used as precursors for the preparation of glycopolymers. Specifically, galactose molecules have great potential in the nanoparticle glycosylation, due to their affinity for expression receptors in cancer cells. On the one hand, galactose-based polymers have been shown to have affinity for the overexpressed asialoglycoprotein (ASGP-R) in liver cancer [30], but also for the prometastatic protein galectin 3 (Gal3) over-expressed in prostate cancer [31], colon [32], breast cancer [33], melanoma [34], multiple myeloma [35] and hemangiosarcoma [36].

**Scheme 1:**
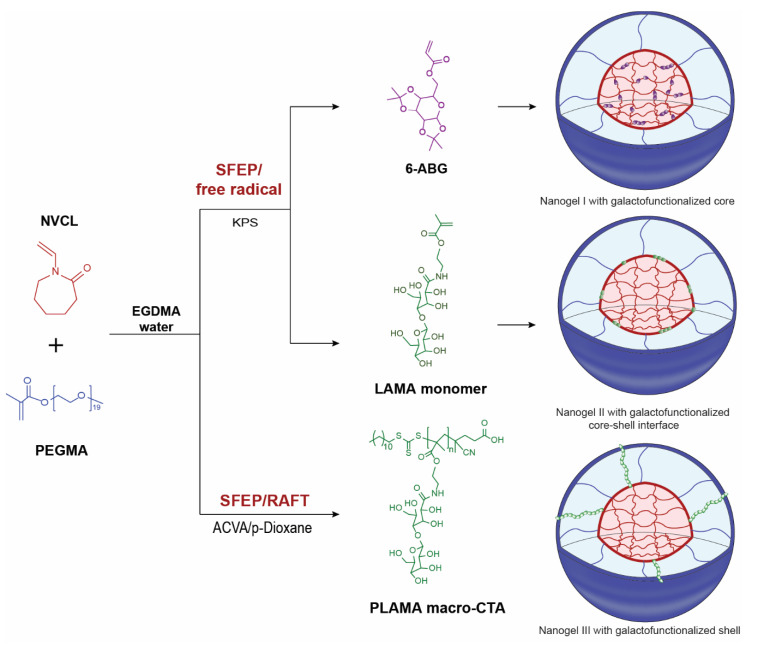
Synthetic strategy for the preparation of PNVCL:PEGMA:GAL core-shell nanogels via SFEP/free radical polymerization (I and II) and via SFEP/RAFT polymerization (III).

Temperature sensitive galactosylated nanoparticles based on poly(*N*-isopropylacrylamide) [37], poly(di(ethyleneglycol) methylether methacrylate) [38], and poly(*N,N*-dimethylaminoethyl methacrylate) [39], have been reported for affinity to ASGP-R receptor. Nevertheless, polymers based on *N*-vinylcaprolactam (NVCL) may become powerful anticancer drug carriers, given that they are also temperature-responsive, in addition to being very stable against hydrolysis, and biocompatible [40]. In previous reports, nanogels based on NVCL have shown to increases their response temperature (T_VPT_) when they were copolymerized with other monomers such as *N*-vinylpyrrolidone or poly(ethyleneglycol) methacrylate (PEGMA) [41,42,43]. In the present work, the synthesis of three families of galactosylated nanogels based on NVCL was studied. The preparation of Nanogels I and nanogels II was attempted by using surfactant free emulsion polymerization (SFEP) via a free-radical mechanism; while the preparation of nanogels III was attempted by SFEP via reversible addition-fragmentation chain transfer (RAFT) polymerization (Scheme 1). The galactose-functionality in the core, at the core-shell interface and at the shell of the nanogels was expected by means of using either a protected GAL-monomer 6-ABG, a GAL-monomer LAMA and a polyLAMA (PLAMA) macro-CTA, respectively. These, three core-shell nanogel types, are proposed as nanomaterials with potential use in the transport of anticancer drugs to target cancer cells due the Gal3-galactan affinity.

## 2. Materials and Methods

### 2.1. Materials

4-Cyano-4(dodecylsulfanylthiocarbonyl)sulfanyl pentanoic acid (the RAFT chain transfer agent or CTA) was synthesized as described in literature [44]. Lactobionic acid (LA, 97%), poly(ethylene glycol) methyl ether methacrylate (PEGMA, M_n_ = 950 g/mol), 4,4′-azobis(4-cyanovaleric acid) (ACVA), potassium persulfate (KPS, 98%), ethylene glycol dimethacrylate (EGDMA, 98%), and 6-O-acryloyl-1,2,:3,4-bis-O-(1-methyl-ethylidene)-α-_D_-galactopiranose (6-ABG, 97%) were used as received. *N*-Vinylcaprolactam (NVCL, 98%) was purified by recrystallization in hexane and dried under vacuum prior to use. All solvents, deuterated and non-deuterated, similarly to all reagents, were purchased from Sigma-Aldrich (Toluca, Mexico); with one exception, ethanol was provided by Fermont (Monterrey, Mexico). Commercial grade distilled water was used (Sparkletts, CA, USA).

### 2.2. Synthesis of PLAMA-Macro CTA

The monomer 2-lactobionamidoethyl methacrylate (LAMA) was synthesized according to previously reported protocols [44]. The synthesis of PLAMA macro-CTA was carried out by RAFT polymerization. A typical synthesis of PLAMA macro-CTA was conducted as follows. 0.5 g LAMA monomer and 0.036 g ACVA were dissolved in 8 mL of H_2_O:EtOH (9:1), simultaneously the CTA (0.145 g) was dissolved in THF (1 mL) and then both were mixed inside an ampoule containing a magnetic stir-bar (Scheme S1 in the Supplementary Materials file:SM). Oxygen was removed of the reaction mixture by freeze-thaw cycles in Argon atmosphere and the ampoule was sealed using a torch. After that, the monomer solution was heated to 70 °C in a preheated oil bath maintaining constant magnetic stirring for 16 and 24 h. After the desired polymerization time the reaction was stopped by cooling with ice-water. Finally, the product was isolated by precipitation in cold acetone, filtered-off and dried in vacuum at room temperature. Two PLAMA macro-CTAs were obtained, one polymerized for 16 h and the second one for 24 h.

### 2.3. Synthesis of PNVCL:PEGMA Galactosilated Nanogels I and II via SFEP/Free Radical Polymerization

Two types of galactosylated PNVCL:PEGMA:GAL nanogels were synthesized by surfactant free emulsion polymerization (SFEP) via free radical mechanism. The reaction was carried out in a schlenk flask containing a magnetic stir-bar connected to a nitrogen inlet using quantities described in Table S1 in SM. In the first one (nanogels I), the protected GAL-monomer 6-O-acryloyl-1,2,:3,4-bis-O-(1-methyl-ethylidene)-α-_D_-galactopiranose (6-ABG) was added to the reaction mixture in water containing NVCL, PEGMA and EGDMA and stirred strongly but avoiding the formation of foam. For the second one (nanogels II), the GAL-monomer LAMA was added instead of 6-ABG. One example follows: *N*-vinylcaprolactam (0.3 g, 2.16 mmol), PEGMA (0.2 g, 0.21 mmol), and EGDMA (12.2 μL, 0.0128 g, 0.065 mmol) were dissolved in 48 mL of deionized water. At the same time, the initiator KPS (0.075 g, 0.277 mmol) was dissolved in 2 mL of deionized water, too. The reaction mixture containing NVCL, PEGMA, EGDMA and LAMA was bubbled with nitrogen for 30 min to remove the oxygen present. Subsequently, the mixture was placed in a preheated oil bath at 85 °C for 20 min under strong stirring; and afterwards, the initiator (KPS) was added to initiate the polymerization reaction (Scheme 1). The reaction was stopped by freezing after 1 h. In all reactions the products were purified via dialysis against distilled water for 5 days, using Spectra/Por dialysis membrane with MWCO = 12000–14000 Da, performing water changes twice daily. Samples were frozen and lyophilized on a Labconco Freeze Dry System Freezone 4.5 (Kansas City, MI, USA). Then, the samples were stored in a refrigerator until use.

### 2.4. Synthesis of PNVCL:PEGMA Galactosilated Nanogels III vía SFEP/RAFT Polymerization

The synthesis of nanogels via SFEP/RAFT polymerization was carried out using quantities described in Table S1 in SM. Water was used as medium of polymerization, primarily. In this way, it can be considered that Nanogel III was prepared similarly to those obtained by SFEP/free radical polymerization, one example follows: *N*-vinylcaprolactam (0.3 g, 2.16 mmol), PEGMA (0.2 g, 0.21 mmol), EGDMA (12.2 μL, 0.0128 g, 0.065 mmol) were dissolved in 48 mL of deionized water, while the PLAMA macro-CTA (0.096 g, 0.053 mmol) and ACVA (0.024 g, 0.086 mmol) were dissolved in 1 mL of *p*-dioxane each. Then the procedure of mixing and degassing was followed as described before. The reaction temperature was 70 °C and was stopped by freezing after 24 h. In all reactions the resulting products were purified and stored similarly to nanogels I and II.

### 2.5. Hydrodynamic Diameter, Sensitivity to Temperature and Zeta Potential

To analyze size distributions of the nanogels obtained, dynamic light scattering (DLS) was used on a Malvern Instruments Zetasizer Nano-ZS (ZEN 3690), (Malvern, Worcestershire, UK) equipped with a red laser (630 nm) and a measurement detector at 90°. For this characterization, purified and re-dispersed materials were used. The concentration employed was 1 wt % of nanogels in deionized water. The size distribution by intensity using CONTIN analysis is reported. The hydrodynamic diameters (D_h_) were calculated using the hydrodynamic radius (R_h_) calculation from the Stokes–Einstein equation for spheres (Equation (1)).

(1) $$R_{h}=\frac{kT}{6\pi \eta_{0}D}$$

where *k* is the Boltzmann constant, *T* is the absolute temperature, *η*_0_ is the solvent viscosity, and *D* is the diffusion coefficient determined from DLS data.

Using the same equipment, the response temperature (T_VPT_) of the nanogels was analyzed by DLS. The effect of temperature on the particle size was studied using dispersions at 1 mg mL^−1^ of nanogels in water while the temperature was varied from 20 to 50 °C with 4 min of equilibration time between each temperature value. The reported T_VPT_ is taken as the minimum of the derivative of dD_h_/dT.

Zeta potential (ζ) was also measured using the same Zetasizer Nano-ZS equipment. Measurements were performed on folded capillary cells at 25 °C adjusting the pH of the medium from 5 to 8. Reported values were the average of three runs.

### 2.6. Molecular Weight, Radius of Gyration, and Second Virial Coefficient

To determine the weight-average molecular weight (*M_w_*), radius of gyration (*R_g_*), and second virial coefficient (*A*_2_), of the nanogels, a static light scattering (SLS) method was used. The equipment employed was a Goniometer from Brookhaven Instruments (Holtsville, NJ, USA) model BI-200SM-E multi-angle DLS/SLS with digital autocorrelator TurboCorr. Concentrations of nanogels in water, for these measurements were 0.1, 0.15, 0.25, 0.35, and 0.5 mg mL^−1^ and the measurement angles were selected from 30° to 150°. The Berry plot algorithm was applied for the evaluation of results, using Equation (2).

(2) $${\left(\frac{KC}{R_{\theta }}\right)}^{0.5}={\left(\frac{1}{M_{w}}\right)}^{0.5}\left(1+\frac{q^{2}R_{g}^{2}}{6}\right)(1+A_{2}M_{w}C)$$

where *K* is an optical constant independent of the concentration of the solution and the molecular weight of the polymer, *C* is the nanogels concentration, *R_ϴ_* is the normalized scattering intensity (Rayleigh ratio) at the scattering angle *θ, M_w_* is the weight-average molecular weight, *R_g_* is the radius of gyration, *A_2_* is the second virial coefficient, and *q* is the magnitude of the scattering vector, expressed as *q* = (4 p*n*/λ_0_)sin(*θ*/2), with *n* and *λ*_0_, being the solvent refractive index, and the laser wavelength, respectively.

### 2.7. Chemical Structure and Composition

Fourier transform infrared spectroscopy (FT-IR) was used to analyze the chemical structure of nanogels using a Spectrum 400, FT-IR/FT-NIR Spectrometer (Perkin Elmer Cetus Instruments, Norwalk, CT, USA) with a diamond attenuated total reflection (ATR) accessory in the spectral range from 4000 to 650 cm^−1^. The samples were used directly as dried powders and 16 scans were collected.

The chemical composition of nanogels was evaluated by hydrogen nuclear magnetic resonance spectroscopy (^1^H-NMR) using a Bruker AVANCE III HD NMR 400 MHz equipment (Billerica, MA, USA). Dried nanogels of known weight (20 mg) were dispersed in deuterated chloroform (CDCl_3_) (600 μL) or dimethyl sulfoxide-d_6_ (DMSO-d_6_) (600 μL) using an ultrasonic bath for 10 min keeping the dispersion cold.

### 2.8. Morphological Analyses

Transmission electron microscopy micrographs (TEM) were acquired using a H7500 transmission electron microscope (Hitachi Co. Ltd., Tokyo, Japan) operating at an accelerating voltage of 80 kV. Samples were prepared by dispersing 0.5 mg of nanogels in 1 mL of water under stirring for 48 h. A drop of those dispersions was poured over a 75-mesh nickel grid coated with a thin layer of carbon/collodion followed by removing excess liquid at room temperature. Afterwards, the samples were stained using a 1% uranyl acetate solution for 1 min.

## 3. Results and Discussion

### 3.1. Synthesis of PLAMA-Macro CTA

**Figure 1:**
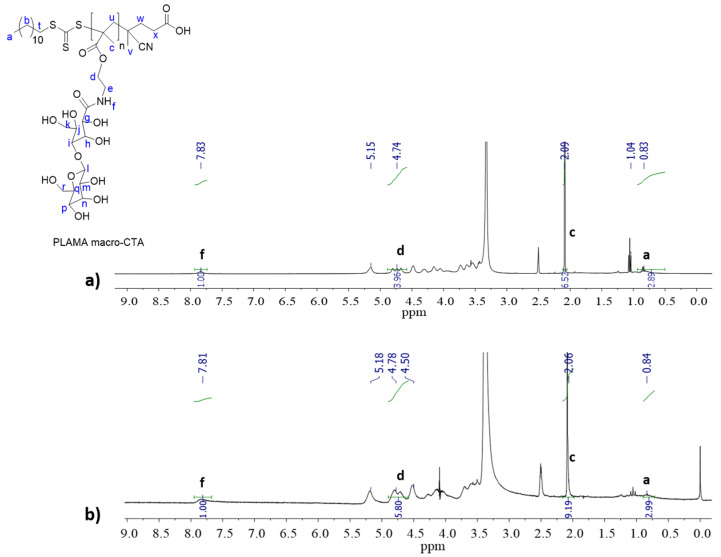
^1^H-NMR spectrum of PLAMA macro-CTA obtained at different polymerization times: (**a**) 16 h with 1342 Da, and (**b**) 24 h with 1812 Da.

The formation of the LAMA monomer was verified by FT-IR and ^1^H-NMR (Figures S1 and S2 in SM), and the product was obtained with a yield above 90%. In the case of PLAMA-macro CTA, both products (obtained after 16 and 24 h of reaction) were pure, as shown in the ^1^H-NMR spectrum (Figure 1). The hydrogen *f* corresponds to the -NH at 7.83 ppm, the signal *d* at 4.74 ppm corresponds to the methylene attached to oxygen of ester of LAMA and the signal *a* at 2.09 ppm corresponds to the methyl hydrogens of LAMA. The signal at 0.86 ppm corresponds to the methyl terminal group of the CTA, with an integration of 3. According to the integrations obtained for each macro-CTA at 0.86 ppm in relation to the signal at 2.09 ppm, it was possible to estimate the molecular weight of the obtained macro-CTAs, being 1342 and 1812 Da for 16 and 24 h of reaction time, respectively.

### 3.2. Synthesis of PNVCL:PEGMA Galactosylated Nanogels via SFEP/Free Radical or SFEP/RAFT Methods

For galactose-functionalized nanogels prepared via SFEP/free radical methods, either 6-ABG (nanogels I) or LAMA (nanogels II) were used as GAL-based monomers, also NVCL, PEGMA and EGDMA were added to the synthesis recipe, while potassium persulfate (KPS) was used as free-radical initiator. On the other hand, the PLAMA macro-CTA was used for the preparation of galactose-functionalized nanogels III via SFEP/RAFT polymerization with NVCL, PEGMA and EGDMA; the free-radical initiator was 4,4,-azo-bis(4-cyano valeric acid) (ACVA). In both types of polymerizations, the reactions were carried out similarly in distilled water. The content of each ingredient in the different synthetic recipes is shown in the Supporting Information file (Table S1 in SM).

In the case of nanogels prepared via SFEP/free radical polymerization, an aqueous system including NVCL, PEGMA and crosslinker EGDMA was used, the GAL moiety was introduced either via protected, less hydrophilic, GAL-monomer 6-ABG (nanogels I) or via water soluble unprotected, highly hydrophilic GAL-monomer LAMA (nanogels II). KPS was used as initiator. A reaction temperature of 85 °C was used; well above the lower critical solution temperature (LCST) of the corresponding linear PNVCL polymer in water. At this temperature, the polymer initially formed, turns hydrophobic but does not precipitates, since it is stabilized by the hydrophilic comonomer PEGMA, forming a stable nucleus progressively crosslinked with EGDMA, leading to nanogel formation. Since LAMA is highly water soluble, it copolymerizes with NVCL but tends to grow in the interface between hydrophobic PNVCL-core and hydrophilic PEGMA-shell; while in the case of 6-ABG being less hydrophilic, it is expected that it is copolymerized with NVCL in the core of the nanogel.

The SFEP/RAFT copolymerization in water allows, theoretically, the preparation of core-shell nanogels, similarly to a SFEP/free-radical mechanism, with controlled size, molecular weight, and responsive behavior. A reaction temperature of 70 °C was used; forming NVCL:PEGMA stable nucleus progressively crosslinked with EGDMA and controlled by the hydrophilic macro-CTA employed, leading to nanogel formation. Since PEGMA contains a polymerizable methacrylate end-group and the PLAMA macro-CTA is hydrophilic, they are chemically attached to the nanohydrogels forming a PEG/GAL shell in nanogels III [42,45]. This strategy to obtain temperature-responsive nanogels takes advantage of the polymerization induced thermal self-assembly and stabilization of the forming nanoparticles [46,47], while the macro-CTA controls the polymerization rate. In all systems, the conversions were determined gravimetrically and resulted to be similar, between 52 and 55% (see data in Table S1 in SM).

**Figure 2:**
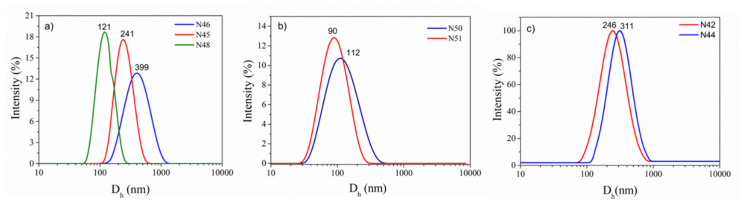
Size distributions by DLS for nanogels: (**a**) NVCL:PEGMA:6-ABG (SFEP/free radical nanogels I, protected GAL); (**b**) NVCL:PEGMA:LAMA (SFEP/free radical nanogels II, GAL), and (**c**). NVCL:PEGMA:PLAMA macro-CTA (SFEP/RAFT nanogels III, GAL macro-CTA).

The nanogel sizes were analyzed by dynamic light scattering (DLS). DLS-data showed unimodal distributions as observed in Figure 2, for nanogels with different content of GAL (6-ABG monomer, LAMA monomer or PLAMA macro-CTA). On the one hand, it is reported in literature that the preparation of core-shell nanogels is well-controlled based on size and polydispersity by surfactant free emulsion polymerization [41,46]. The SFEP/free radical method used in this report was adapted from previous reports on PNVCL nanogels [41,42]. Two groups of nanogels by SFEP/free radical polymerization were synthesized: In the first one, a protected GAL-monomer (6-ABG) was used, to form nanogels with a core containing galactose molecules; while in the second one, a free galactose (LAMA) monomer was added to the reaction mixture, to obtain a GAL-functionalized interface between core and shell. The nomenclature assigned for the nanogels I group was: NVCL_37_._5wt%_:PEGMA_25wt%_:6-ABG_37_._5wt%_ (N46), NVCL_46wt%_:PEGMA_31wt%_:6-ABG_23wt%_ (N45) and NVCL_52wt%_:PEGMA_35wt%_:6-ABG_13wt%_ (N48); and for the nanogels II group was: NVCL_46wt%_:PEGMA_31wt%_:LAMA_23wt%_ (N50) and NVCL_51wt%_:PEGMA_34wt%_:LAMA_15wt%_ (N51).

The size of nanogels by SFEP was analyzed by DLS, and narrow unimodal distributions were obtained. For the first group of nanogels (galactosylated core) the higher content of 6-ABG monomer the bigger hydrodynamic diameter was obtained. It has been reported that for SFEP using PEGMA as a polymerizable stabilizer, the lower the amount of PEGMA, the higher the nanogels diameter obtained [46]. In our case, this behavior is also present, since the higher content on GAL monomer concomitantly leads to a lower amount of PEGMA stabilizer in the synthetic recipe resulting in nanogels with larger size. Nanogels I with 13, 23 and 37.5 wt % of 6-ABG monomer in the reaction mixture, showed D_h_ values of 121, 241 and 399 nm, respectively (Figure 2a); while nanogels II with 23% and 15% of LAMA monomer the diameters measured were 112 and 90 nm (Figure 2b). In the case of the second group (galactosylated core-shell interface), the results showed a noticeable trend in size growth by increasing the amount of LAMA monomer in the reaction; this can be attributed to the formation of hydrogen bonds between these monomers and the dispersing water, which causes the nanogels to be mostly swollen. Furthermore, as the weight fraction of GAL monomer increased, the growing polymer chains become more hydrophilic during the polymerization; thus, bigger hydrophobic cores formed by PNVCL, could be stabilized easily with the poly(LAMA-*co*-PEGMA) hydrophilic chains [48,49].

In the case of SFEP/RAFT nanogels III, it can also be observed that the increase in the GAL concentration from 15 to 23 wt %, results in smaller hydrodynamic diameters (D_h_) (311 to 246 nm, respectively) (Figure 2c). Concomitantly, the polydispersity calculated from the cumulant method (PDI = μ_2_/Г^2^), increases while increasing the GAL (PLAMA macro-CTA) concentration from 0.18 to 0.33 for 15 to 23 wt % of GAL in the feed. Via SFEP/RAFT, the nomenclature used was specified according to the feed as follows: NVCL_46wt%:_PEGMA_31wt%:_GAL_23wt%_ (N42), and NVCL_51wt%_:PEGMA_34wt%_:GAL_15wt%_ (N44), where the GAL content evolves from the PLAMA macro-CTA used in different amounts. An explanation for this behavior can be found if the PLAMA-macro-CTA is considered as an additional stabilizer to the PEGMA in the synthetic recipe for the growing PNVCL block forming the core. A higher amount of stabilizer to a lower core-forming monomer amount result in an overall smaller nanogel.

**Figure 3:**
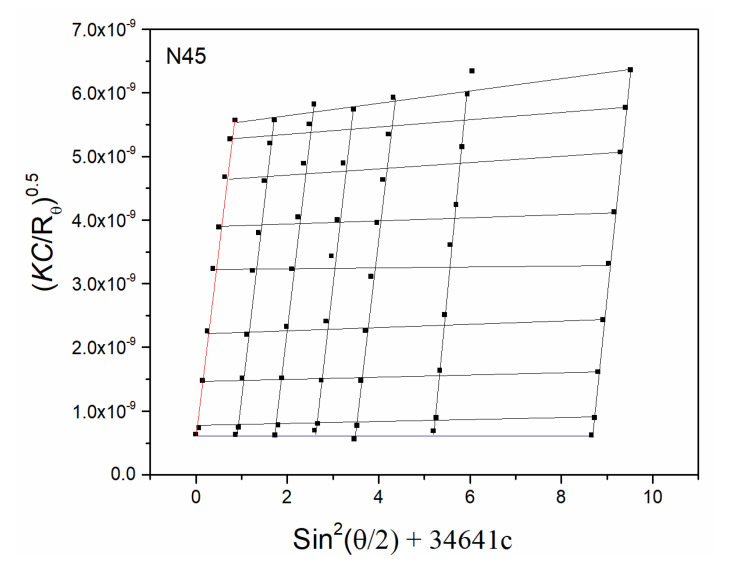
Berry plot of nanogel N45 obtained by SLS.

Molecular weight (M_w_), radius of gyration (R_g_) and the second virial coefficient (A_2_) data, were determined by static light scattering (SLS). The Berry plot for sample N45, measured in water at 25 °C (Figure 3), showed a linear dependence on the scattering angle, and was related to homogeneous particles [50,51]. Data from SLS and DLS are summarized in Table 1 (Figures S3–S9 in SM). Nanogels show M_W_ values between 3.65 × 10^6^ and 2.20 × 10^10^ g mol^−1^ and R_g_ values between 44 and 210 nm. Values of A_2_ are between 2.01 × 10^−9^ and 1.18 × 10^−4^ and indicate a good interaction between nanogels and water. The shape factor, ρ-parameter, was calculated by the ratio between R_g_ and R_h_ (Table 1). The values obtained, show microgel/hard sphere morphology for nanogels N42 and N50, soft sphere for N44, N32 and N51; and star type morphology for N46, N45 and N48, in accordance to values reported by W. Burchard [52]. The nanogels N46, N45 and N48 are the ones prepared using the GAL protected monomer. The ρ-parameter results suggests that, despite the actual size of the products, nanogels prepared with this comonomer (6-ABG) incorporate the GAL-protected moiety in the core statistically mixed with NVCL polymers, while PEGMA chains are presumably located in a stabilizing shell.

**Figure 4:**
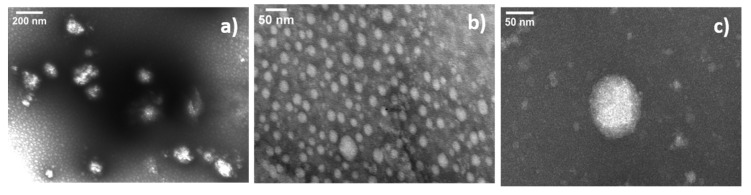
TEM micrographs of selected nanogels: (**a**) N46 (SFEP/free radical, 6-ABG); (**b**) N51 (SFEP/free radical, LAMA); (**c**) N44(SFEP/RAFT, PLAMA).

Figure 4 shows TEM-micrographs of one example of each type of synthesized nanogels. Despite the differences in sizes, it is clear that the shape of the nanogels is more irregular in those synthesized using the protected GAL-monomer (6-ABG); coincidently with the light scattering results of ρ-parameter. Additional TEM-micrographs can be found in the Supporting Information file (Figures S10–S14 in SM).

**Figure 5:**
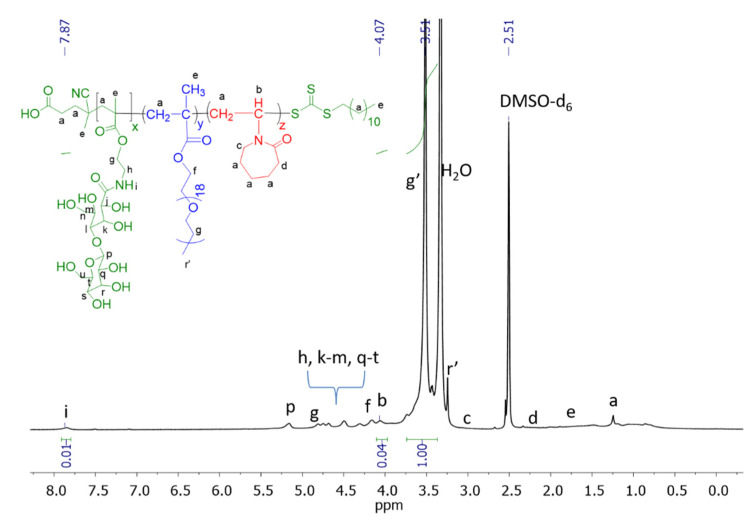
^1^H-NMR spectrum of galactosylated nanogel N42 by RAFT.

The actual content on NVCL, PEGMA and GAL in the respective synthesized nanogels was determined by hydrogen nuclear magnetic resonance (^1^H-NMR). Figure 5 shows the ^1^H-NMR spectrum of N42 as an example. The signal at 3.51 ppm is assigned to the –CH_2_CH_2_‒O (g’) of PEGMA lateral chain; the signal at 4.15 ppm corresponds to –CH‒ (b) of the backbone of the PNVCL; the signal at 7.85 ppm is assigned to N‒H (i) of the PLAMA macro-CTA. The composition was calculated by integration of signals at chemical shifts of 3.51 pm (PEGMA), 4.15 ppm (NVCL) and 7.85 ppm (macro-CTA, LAMA). ^1^H-NMR spectra of the other nanogels are included in the Supplementary information file (Figures S15–S21 in SM). Table 2 summarizes the compositions of nanogels, which proofs that PEGMA, NVCL and GAL are present in the produced nanogels structure. In general, NVCL content in products is lower than in the feed, while PEGMA and GAL content is higher than expected; since data indicated a preferred incorporation of comonomers over NVCL in the polymer network. This is a result of the different polymerization kinetics of NVCL and the other comonomers. NVCL is a monomer of the family of vinylamides, similar to vinylacetate (VAc), and its polymerization is rapid if initiated by radicals. However, in radical copolymerization, its propagating radical is not stable.

Therefore, if the second comonomer forms a more stable propagating specie, e.g., methacrylate radical, then NVCL will copolymerize sluggishly, favoring the incorporation of a more stable (methacrylate) comonomer [40]. Although, there are no reports on copolymerization parameters of NVCL and PEGMA in water, which could explain the copolymerization behavior, there is a report on NVCL with methyl methacrylate (MMA) prepared by microemulsion; the copolymerization parameters are r_1_ = 0.25 (for NVCL) and r_2_ = 2.63 (for MMA) evidencing the mismatch in copolymerization reactivity leading to lower amounts of NVCL in the copolymers than in the feed [53]. As a conclusion, it is not surprising that in our case, the NVCL monomer is incorporated in all three cases in lower amount than in the feed given the kinetics of NVCL copolymerization with methacrylates. The amount of GAL moieties in the nanogels prepared, from 5.7% to 28% is in the same order of magnitude as in other reports on galactose functionalized nanogels in literature [37] and most likely is enough for targeting purposes.

**Figure 6:**
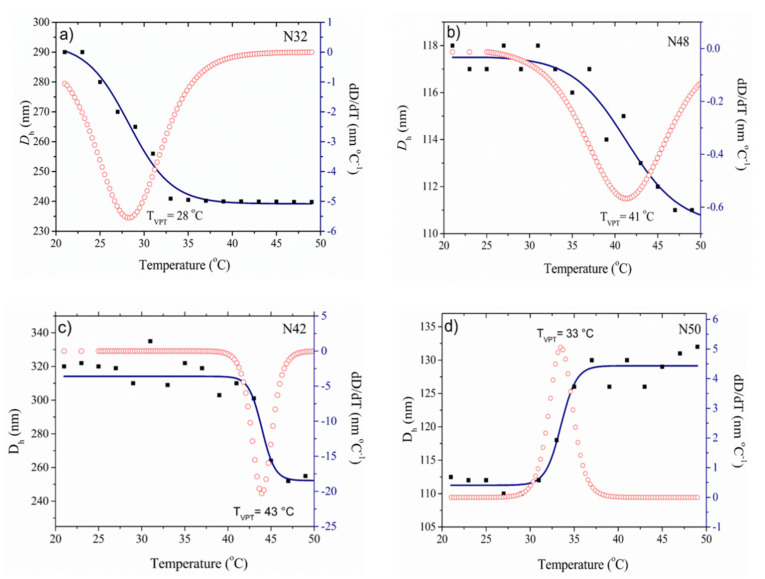
Nanogels size analysis as a function of temperature by DLS: (**a**) NVCL_60wt%_:PEGMA_40wt%_ (N32), (**b**) NVCL_52wt%_:PEGMA_35wt%_:6-ABG_13wt%_ (N48), (**c**) NVCL_46wt%_:PEGMA_31wt%_:GAL_23wt%_ (N42), (**d**) NVCL_46wt%_-PEGMA_31wt%_-LAMA_23wt%_ (N50).

It was important to determine the effect of incorporation of the GAL comonomer in the nanogels thermosensitive behavior. The effect of temperature on the galactosylated nanohydrogel size dispersed in water was analyzed by DLS. Usually, temperature sensitive polymer networks based on LCST/polymers are swollen below the volume phase transition temperature (T_VPT_) and shrink above it. The T_VPT_ was estimated from the first derivative of hydrodynamic diameter measured at different temperatures. The results for some nanogels are shown in Figure 6. A nanogel without GAL monomer (nanogel N32) serves as a blank nanomaterial for comparison purposes (Figure 6a), this nanogel shows a transition temperature at 28 °C, a temperature too low for biomedical applications. In the case of the SFEP/RAFT nanogels containing GAL, N44 (Figure S22 in SM) and N42 (Figure 6c), they showed an increase on the T_VPT_ values from 40 to 43 °C, as the concentration of the galactose PLAMA macro-CTA increases from 12% to 18.9 mol%. It is well known that for thermoresponsive copolymers showing a LCST, the presence of a hydrophilic comonomer can increase the T_VPT_, while the increase of concentration of the thermosensitive monomer decrease the T_VPT_ to the homopolymer’s original value [54]. This phenomenon can be attributed once again to the formation of hydrogen bonds between GAL and the environment (water), requiring a higher energy (temperature) to break those interactions and induce a coil to globule transition of the individual chains, resulting in shrinkage of the nanogels.

In the family of SFEP nanogels containing protected GAL-monomer (galactosylated core), N48 (Figure 6b), N45 (Figure S23 in SM) and N46 (Figure S24 in SM), the transition temperature shows values of 41, 44 and 46 °C, respectively, with increase in content on the protected GAL-monomer (6-ABG) of 23.8, 27.2 and 28.1 mol%. These results can be attributed to the effect to mixing the protected GAL-monomer and *N*-vinylcaprolactam in the nanogel core. Despite being 6-ABG less hydrophilic in comparison to LAMA, the increase in T_VPT_ suggests that more energy is needed to trigger the shrinkage of this type of nanogels I, due to the relative large amount on this GAL-monomer, more than 20 mol% in the nanogels as compared to less than 8 mol% in the case of LAMA (see composition data in Table 2). On the other hand, nanogels synthesized with LAMA monomer (galactosylated interface), N50 (Figure 6d) and N51 (Figure S25 in SM), showed an aggregation behavior opposed to shrinkage; the first one with a small increase in size of 20 nm approximately, while the nanogel N51 aggregates to very large microparticles (1200 nm approximately). Values of T_VPT_ were 33 and 32 °C respectively. It is an interesting observation that these nanogels were the smallest prepared (radii below 60 nm), with the lowest content on GAL monomer from 5.7 to 7.7 mol% (Table 2). Since the thermosensitivity of PNVCL is related to a phase transition of polymer chains from soluble to non-soluble in water, it is not surprising that aggregates are formed by heating. Cases where the nanogels shrink without aggregation, is attributed to the shell of the nanogels, which has a moderate shielding effect to prevent it. It is reported in literature that aggregation of lightly crosslinked NVCL-nanogels is not uncommon [10].

**Figure 7:**
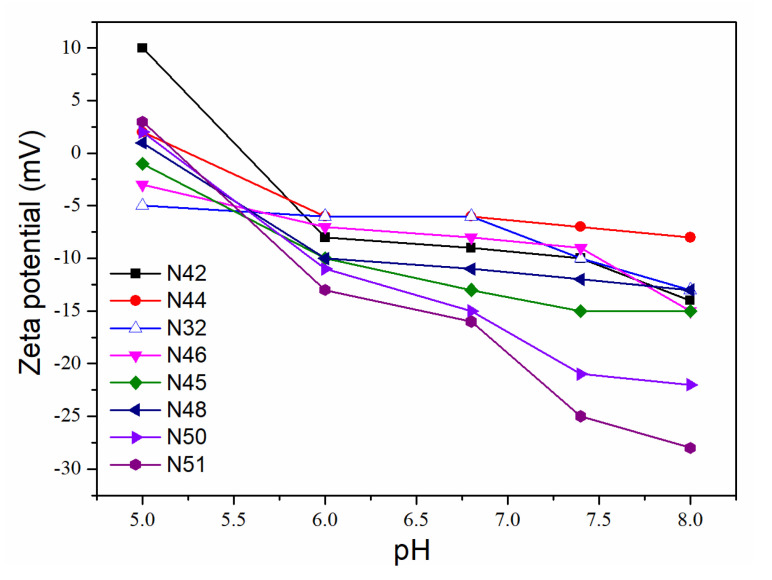
Zeta potential of nanogels at different pH-values.

The surface charge of the nanogels was estimated through the zeta potential by DLS. Figure 7 shows the pH dependence of zeta potential for the synthesized nanogels. All nanogels show a negative zeta potential at pH ≥ 6, while nanogels N32, N46 and N45 show negative zeta potential even at pH 5. It is important to mention that the free-radical initiators used for nanogels preparation have acid groups that are ionized at pH values ≥ 5. These groups remain attached to the nanogels surface after initiation of polymerization. Furthermore, the presence of a reducing sugar in galactose monomers may influence the surface charge of the nanogel. Otherwise stated, by increasing the pH of the dispersion, the sugars are mostly in their reducing form, which also implies a greater presence of -OH groups. Nanogels N42, N44, N48, N50 and N51 have their isoelectric point (no charge) between pH 5 and 6. The results indicate that the hydrophilic chains containing GAL and PEGMA stabilize the nanogels in aqueous solution at room temperature. Similar results, where the zeta potential decreases to negative values while the pH increases, have been observed also in nanometric systems with other oligosaccharides [55,56,57]. The fact that these nanogels are anionic at physiological pH may be advantageous for its application in drug delivery systems since a cationic charge at pH 7.4 is usually related to cytotoxicity toward cell-lines [58].

## 4. Conclusions

A series of nanogels with cross-linked PNVCL core and a shell of PEGMA and galactofunctionalized core, shell or interface, were prepared in water via SFEP using a free radical or RAFT polymerization mechanism. The incorporation of a GAL monomer (LAMA macro-CTA by SFEP/RAFT; and LAMA/6-ABG monomers by SFEP/free radical) allowed to increase the transition temperature of PNVCL/PEGMA nanogels from 28 °C to values around the relevant physiological temperatures (37–41 °C). In general, the nanogels obtained, possess spheroidal shape with diameters between 90 and 370 nm in their swollen state and GAL-content from 5 to 28 wt%. Nanogels I, containing higher amounts of protected GAL-moieties in the core resemble a hyperbranched semi-spherical particle, according to DLS results, and showed the highest change in T_VPT_ with GAL content. Nanogels II containing less GAL moieties in the core/shell interface resemble a spherical core-shell particle with less impact on its T_VPT_ values. Nanogels III containing intermediate amounts of GAL-moieties, but in this case as chains in the shell, also resemble a spherical core-shell particle, while their impact on the T_VPT_ is also intermediate. These materials have a great potential for drug delivery in cancer treatment, due to their temperature response, small sizes, potential non-cytotoxicity evolving from its constituents, and the potential recognition of cancer cells via the Galactan-Galectin 3 interaction. The effect of location of the GAL moiety in the nanogels towards the recognition of Galectin 3, remains as a goal for future studies.

## Figures and Tables

**Table 1:** Light scattering data of nanogels prepared by SFEP (free radical and RAFT).

Nanogel	R_h_^a^ (nm)	PDI ^a^	M_w_^b^ (g mol^−1^)	R_g_^b^ (nm)	A_2_^b^ (mol mL g^−2^)	ρ-Parameter ^c^
Nanogels I						
N46	185	0.14	2.20 × 10^10^	210	3.75 × 10^−7^	1.13
N45	120	0.11	1.57 × 10^9^	173	2.01 × 10^−9^	1.44
N48	61	0.08	8.74 × 10^8^	76	4.83 × 10^−7^	1.24
Nanogels II						
N32	145	0.37	1.11 × 10^8^	143	2.64 × 10^−6^	0.98
N50	56	0.19	3.65 × 10^6^	44	4.12 × 10^−6^	0.78
N51	45	0.18	3.53 × 10^6^	43	4.01 × 10^−6^	0.95
Nanogels III						
N42	144	0.33	9.10 × 10^6^	112	1.18 × 10^−4^	0.78
N44	156	0.18	7.90 × 10^7^	154	2.50 × 10^−6^	0.98

^a^ Data obtained by DLS. ^b^ Data of nanogels obtained by SLS in water at 25 °C. ^c^ Ratio R_g_/R_h_.

**Table 2:** Composition of nanogels prepared by SFEP (free radical and RAFT) as determined by ^1^H-NMR.

Nanogel	Feed Composition (mol%) ^a^			Product Composition (mol%) ^a^		
	NVCL	PEGMA	GAL	NVCL	PEGMA	GAL
Nanogels I						
N46	66.7	5.5	27.8	65.5	6.5	28.1
N45	76.9	6.3	16.8	65.3	7.6	27.2
N48	83.9	6.9	9.2	59.6	16.6	23.8
Nanogels II						
N32	92.2	7.8	0	92	8	0
N50	80.3	7.8	11.9	65.4	26.9	7.7
N51	85.4	8.3	6.3	67.9	26.4	5.7
Nanogels III						
N42	86.0	10.3	3.7	56.6	24.5	18.9
N44	88.3	8.4	3.3	76	12	12

^a^ The EGDMA content was not considered.

## Supplementary Materials

The following are available online at https://www.mdpi.com/2073-4360/12/9/2150/s1, Figure S1: FT-IR spectra of lactobionic acid and LAMA monomer, Figure S2: ^1^H-NMR spectrum of LAMA monomer, Figure S3: Berry plot by SLS analysis of nanogel N42, Figure S4: Berry plot by SLS analysis of nanogel N44, Figure S5: Berry plot by SLS analysis of nanogel N32, Figure S6: Berry plot by SLS analysis of nanogel N50, Figure S7: Berry plot by SLS analysis of nanogel N51, Figure S8: Berry plot by SLS analysis of nanogel N46, Figure S9: Berry plot by SLS analysis of nanogel N48, Figure S10: TEM micrograph of nanogel N42 taken at 80 KeV, Figure S11: TEM-micrograph of nanogels N32 taken at 80 KeV, Figure S12: TEM micrograph of nanogels N50 taken at 80 KeV, Figure S13: TEM-micrograph of nanogels N48 taken at 80 KeV, Figure S14: TEM micrograph of nanogels N45 taken at 80 KeV, Figure S15: ^1^H-NMR of nanogel N32, Figure S16: ^1^H-NMR of nanogel N44, Figure S17: ^1^H-NMR of nanogel N46, Figure S18: ^1^H-NMR of nanogel N45, Figure S19: ^1^H-NMR of nanogel N48, Figure S20: ^1^H-NMR of nanogel N50, Figure S21: ^1^H-NMR of nanogel N51, Figure S22: D_h_ of nanogel N44 as function of temperature obtained by DLS, Figure S23: D_h_ of nanogel N45 as function of temperature obtained by DLS, Figure S24: D_h_ of nanogel N46 as function of temperature obtained by DLS, Figure S25: D_h_ of nanogel N51 as function of temperature obtained by DLS, Scheme S1: Synthesis of PLAMA macro-CTA, Table S1: Reaction conditions for the preparation of nanogels NVCL:PEGMA:GAL using 3 mol% of EGDMA with respect to NVCL as crosslinker.
