# Supplementary material for: Different Strategies for the Preparation of Galactose-Functionalized Thermo-Responsive Nanogels with Potential as Smart Drug Delivery Systems
Source: Polymers (Basel). 2020 Sep 21;12(9):2150. doi: 10.3390/polym12092150 (PMC7569999; doi:10.3390/polym12092150)
Supplement: Supplementary file 1 [file polymers-12-02150-s001.pdf]

Supplementary Materials

Different Strategies for the Preparation of Galactose-Functionalized Thermo-Responsive Nanogels with Potential as Smart Drug Delivery Systems

Mirian A. González-Ayón[1], Angel Licea-Claverie[1]*and J. Adriana Sañudo-Barajas[2]

[1]Centro de Graduados e Investigación en Química, Tecnológico Nacional de México/Instituto Tecnológico de Tijuana, Apartado Postal 1166, Tijuana, B.C., C.P. 22454, México.

[2]Centro de Investigación en Alimentación y Desarrollo, A. C. Carretera a El dorado Km 5.5, Culiacán, Sinaloa, C.P. 80110, México.

*Corresponding author: E-mail: aliceac@tectijuana.mx.

Experimental Section

Synthesis of 2-lactobionamidoethyl methacrylate (LAMA)

The formation of the LAMA monomer was verified by signal comparison of LAMA with lactobionic acid by FT-IR. In the lactobionic acid a signal at 3328 cm^{-1} corresponding to O-H stretching vibration is observed, a signal of -CH_2- is present at 2906 cm^{-1}, the C=O stretching band appears at 1736 cm^{-1} for the acid and at 1030 cm^{-1} the –C-O- band from the aliphatic chain, is observed. The FT-IR spectrum of the LAMA monomer shows that the C=O band is displaced to 1708 cm^{-1}, which corresponds to an ester carbonyl, the signal =CH_2 appears at 1644 cm^{-1} and the -NH bending at 1538 cm^{-1} (**Figure S1**), thus confirming the success of the LAMA monomer synthesis. By ^{1}H-NMR (**Figure S2**), the formation and purity of LAMA was confirmed. In further detail, the signal corresponding to -NH at 7.76 ppm integrates for one hydrogen, at 6.05 and 5.66 ppm, the hydrogens of the vinyl group are shown, the signals of methines *i* and *l* attached to the oxygen that unites the galactose ring to the monomer, appear at 5.18 and 5.10 ppm (both integrate for one hydrogen), methylene *d* attached to the ester of methacrylate is observed at 4.72 ppm with an integration of 2, while methine *g* in the lactobionic acid residue is observed at 4.57 ppm integrating for one hydrogen, the signals of the hydrogens marked as *e, h-k, m-r* that correspond to the majority of the lactobionic acid skeleton hydrogens, are observed between 4.38 and 3.3 ppm; and a signal of methyl *c* (of methacrylate) is observed as a triplet at 1.85 ppm, integrating for 3 hydrogens.

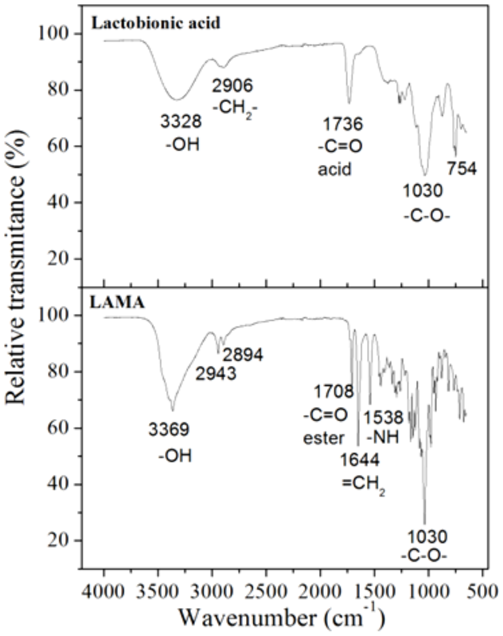

Figure S1. FT-IR spectra of lactobionic acid and LAMA monomer.

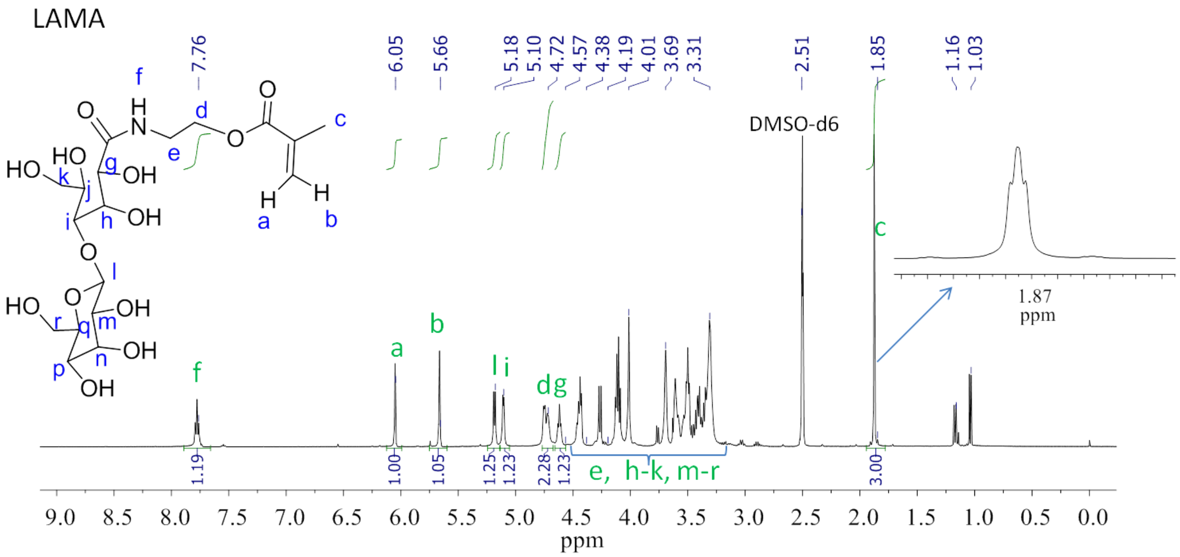

Figure S2. ^{1}H-NMR spectrum of LAMA monomer.

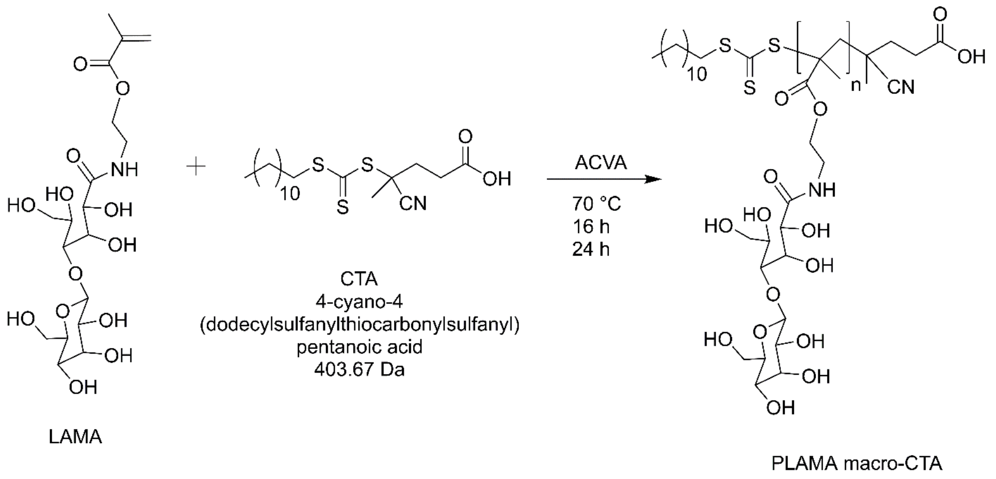

Scheme S1. Synthesis of PLAMA macro-CTA.

Table S1. Reaction conditions for the preparation of nanogels PNVCL:PEGMA:GAL using 3 mol% of EGDMA with respect to NVCL as crosslinker by SFEP (free radical and RAFT).

Nanogel	NVCL:PEGMA:GAL (wt%)	NVCL (g)	PEGMA (g)	GAL (g)	Initiator (g)	Yield (%)[f]
**Nanogels I**	1 h at 85 °C					
N46(6-ABG)	37.5:25:37.5	0.3	0.2	0.30[a]	0.075[d]	52
N45(6-ABG)	46:31:23	0.3	0.2	0.15[a]	0.038[d]	55
N48(6-ABG)	52:35:13	0.3	0.2	0.075[a]	0.019[d]	52
**Nanogels II**	1 h at 85 °C					
N32	60:40:00	0.3	0.2	-	0.024[d]	53
N50(LAMA)	46:31:23	0.3	0.2	0.15[b]	0.038[d]	54
N51(LAMA)	52:35:13	0.3	0.2	0.075[b]	0.038[d]	53
**Nanogels III**	24 h at 70 °C					
N42	46:31:23	0.3	0.2	0.15[c]	0.038[e]	51
N44	51:34:15	0.3	0.2	0.09[c]	0.023[e]	53

[a]6-ABG; [b]LAMA; [c]PLAMA macro-CTA; [d]KPS; [e]ACVA; [f]mass yield

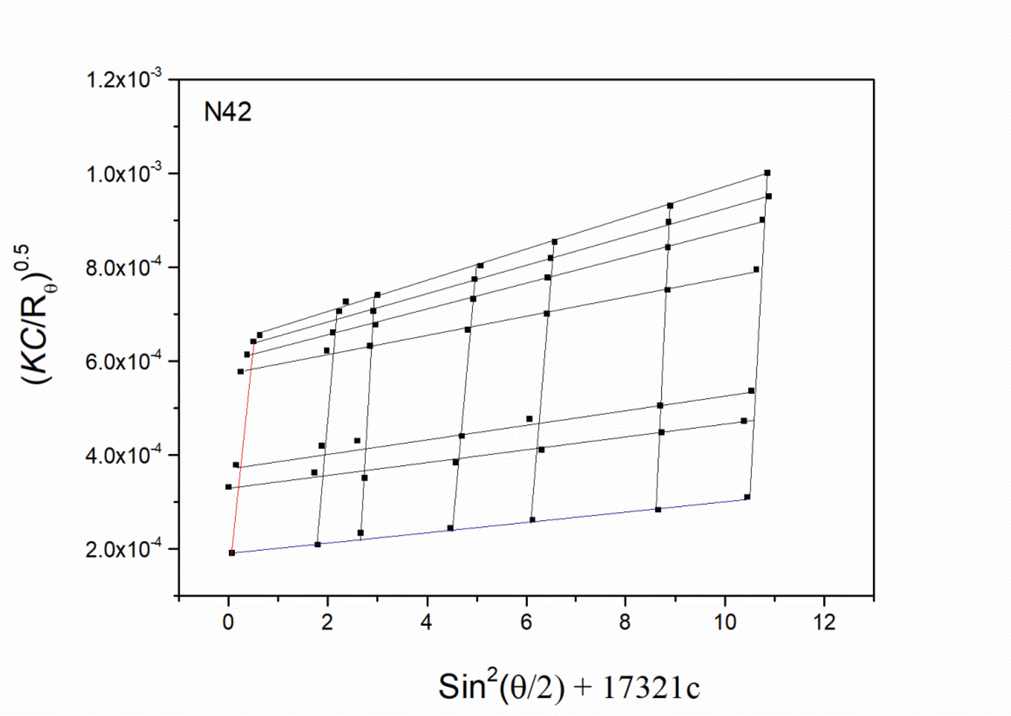

Figure S3. Berry plot by SLS analysis of nanogel N42.

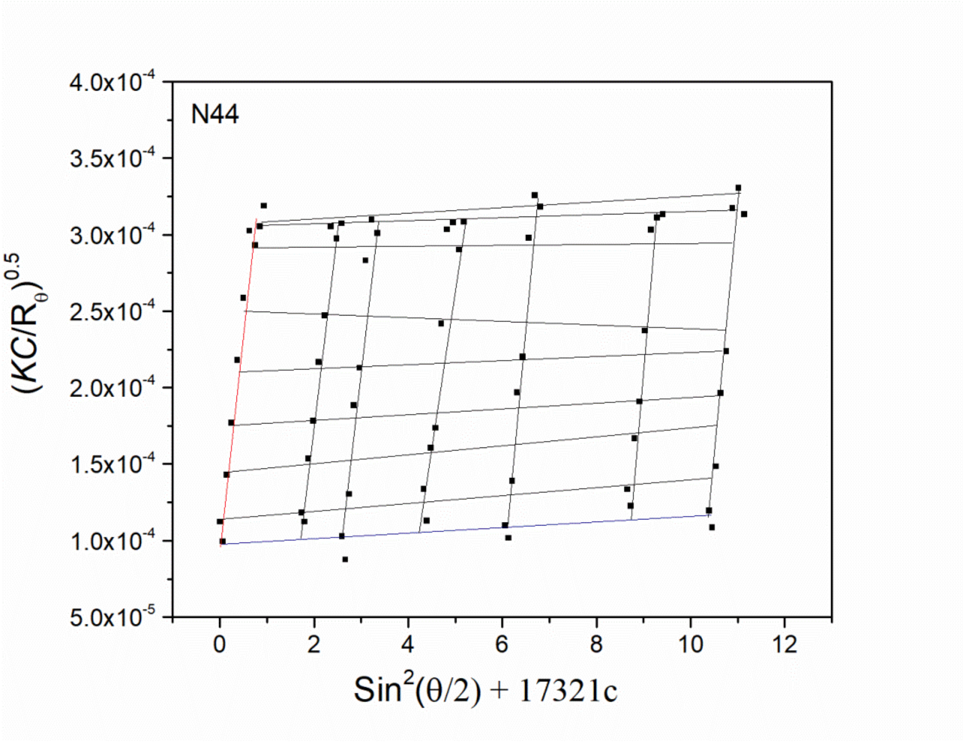

Figure S4. Berry plot by SLS analysis of nanogel N44.

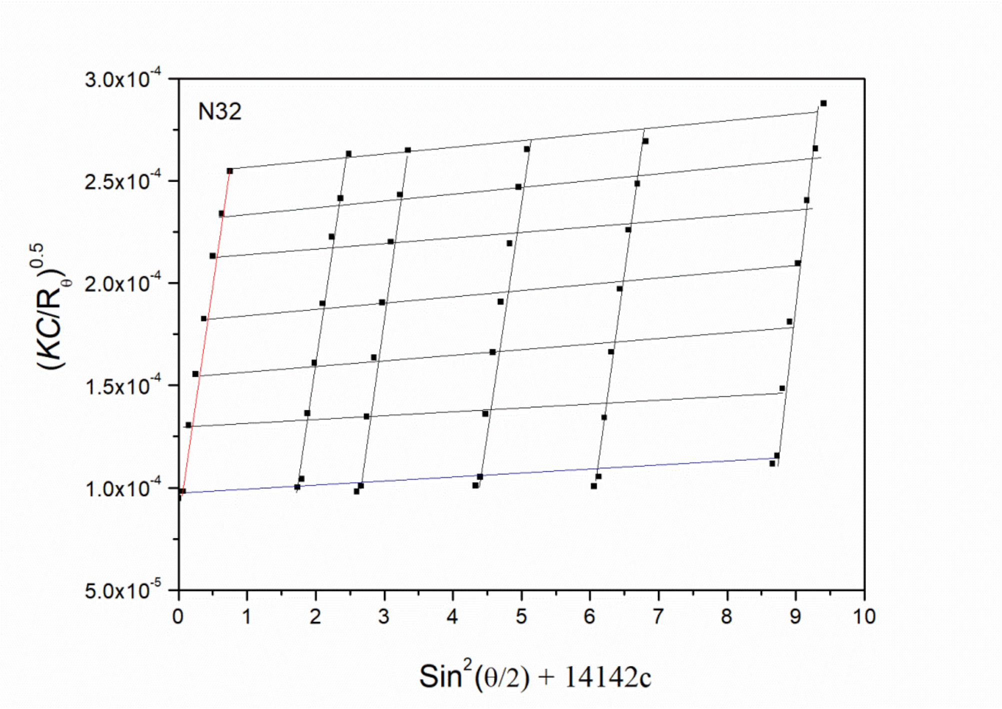

Figure S5. Berry plot by SLS analysis of nanogel N32.

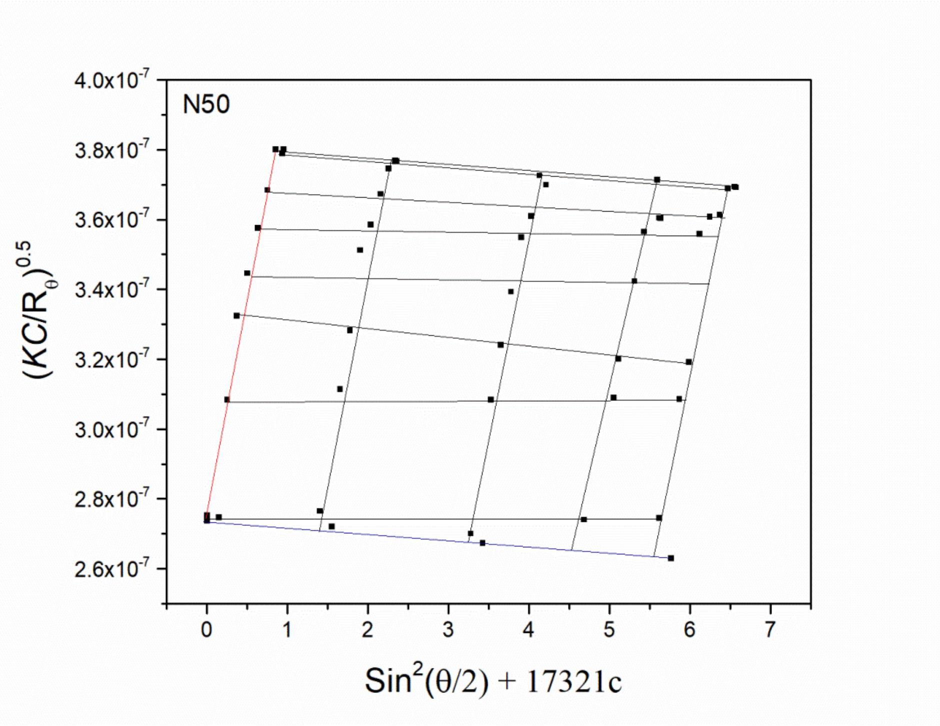

Figure S6. Berry plot by SLS analysis of nanogel N50.

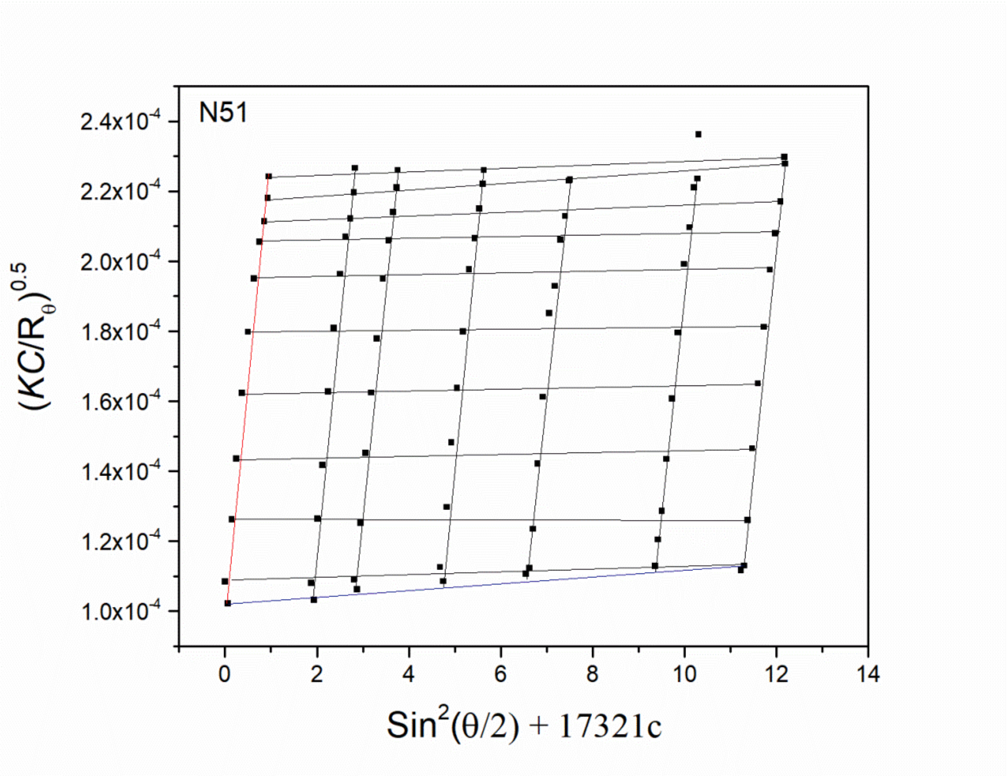

Figure S7. Berry plot by SLS analysis of nanogel N51.

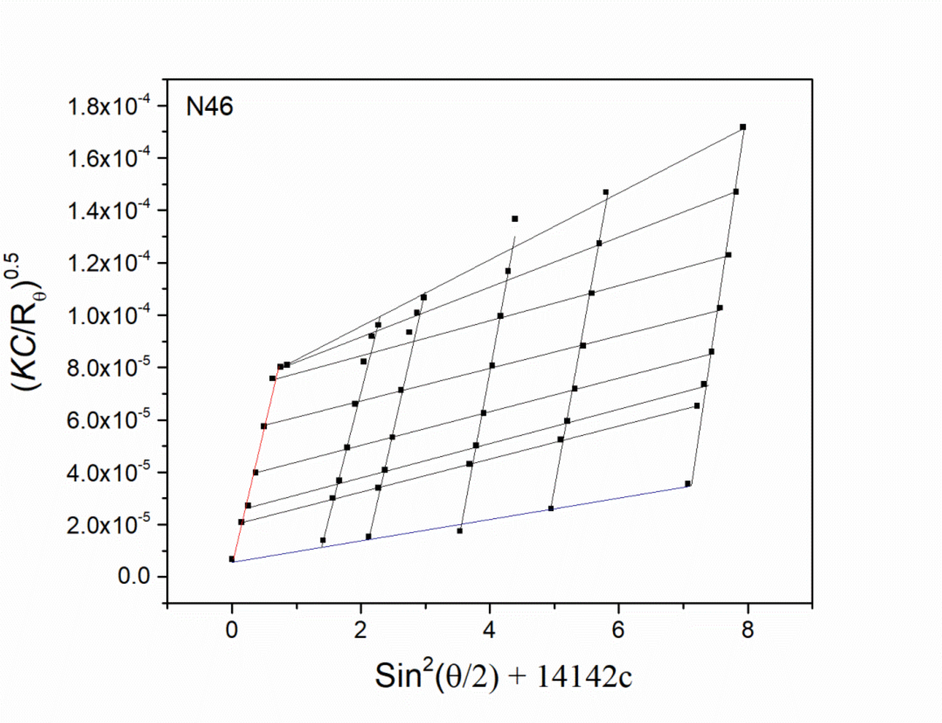

Figure S8. Berry plot by SLS analysis of nanogel N46.

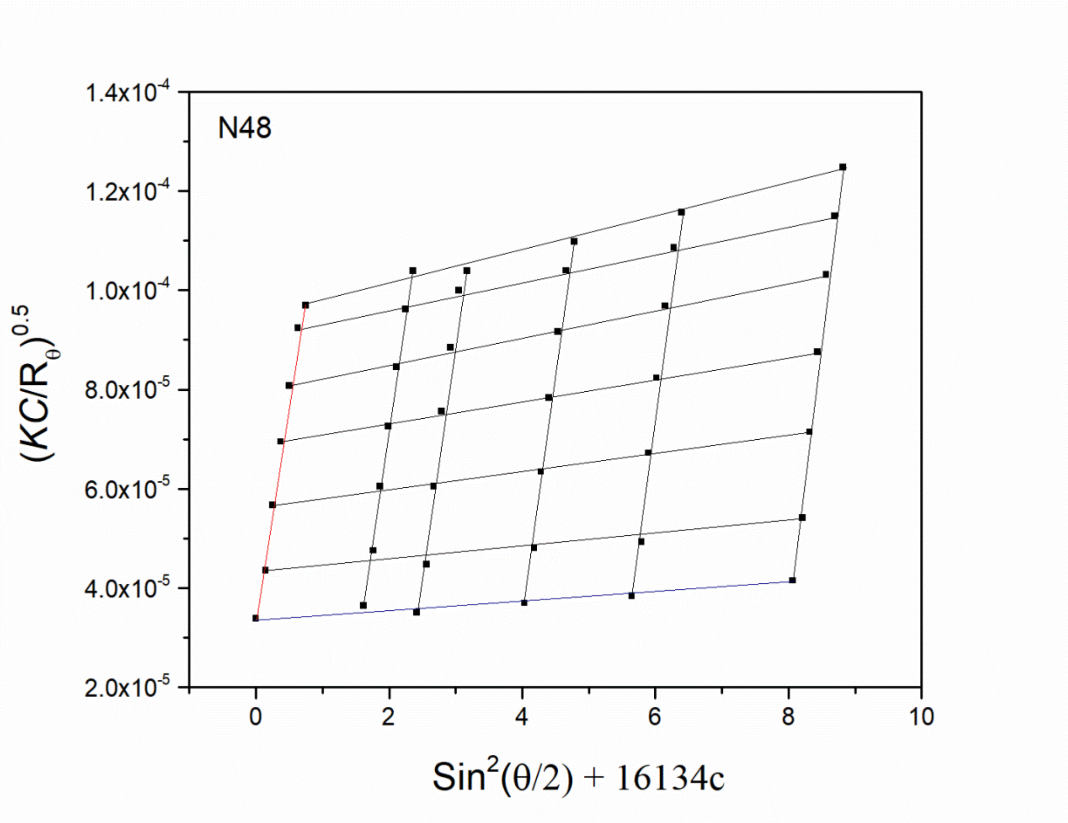

Figure S9. Berry plot by SLS analysis of nanogel N48.

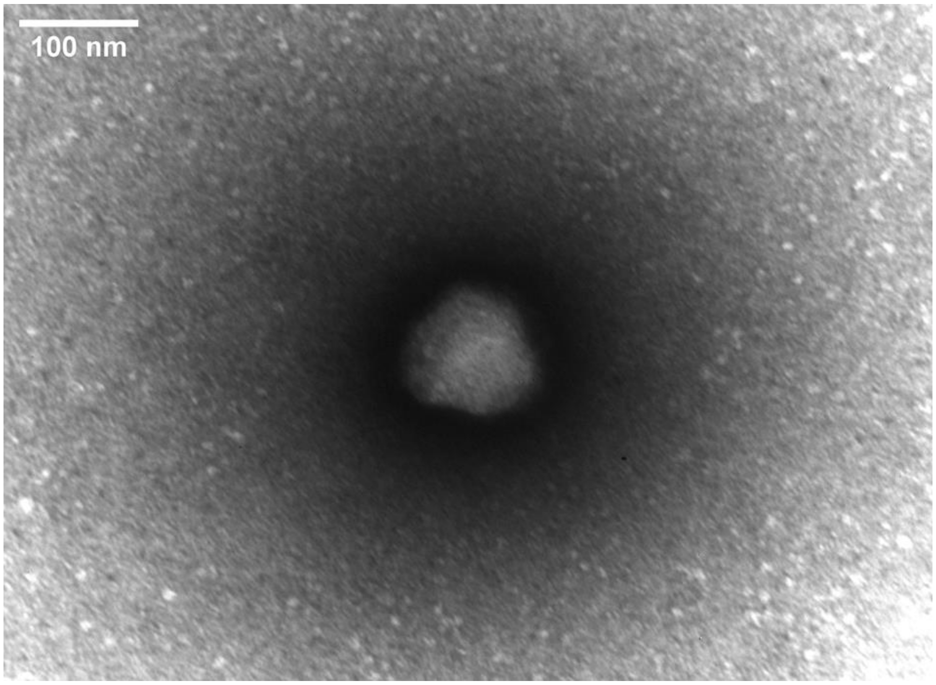

Figure S10 TEM micrograph of nanogel N42 taken at 80 KeV.

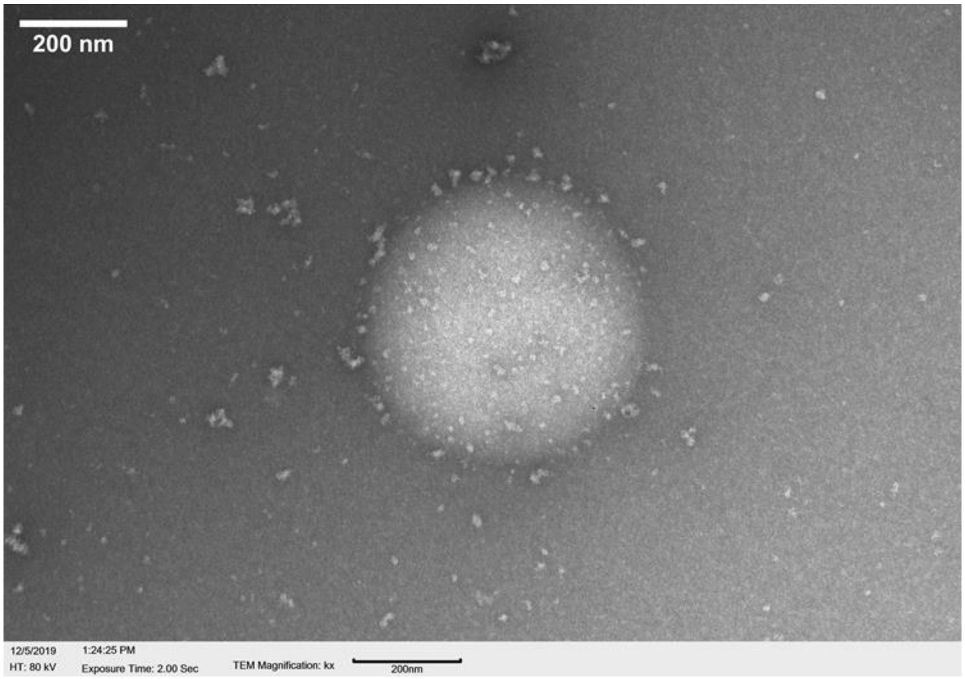

Figure S11: TEM-micrograph of nanogels N32 taken at 80 KeV.

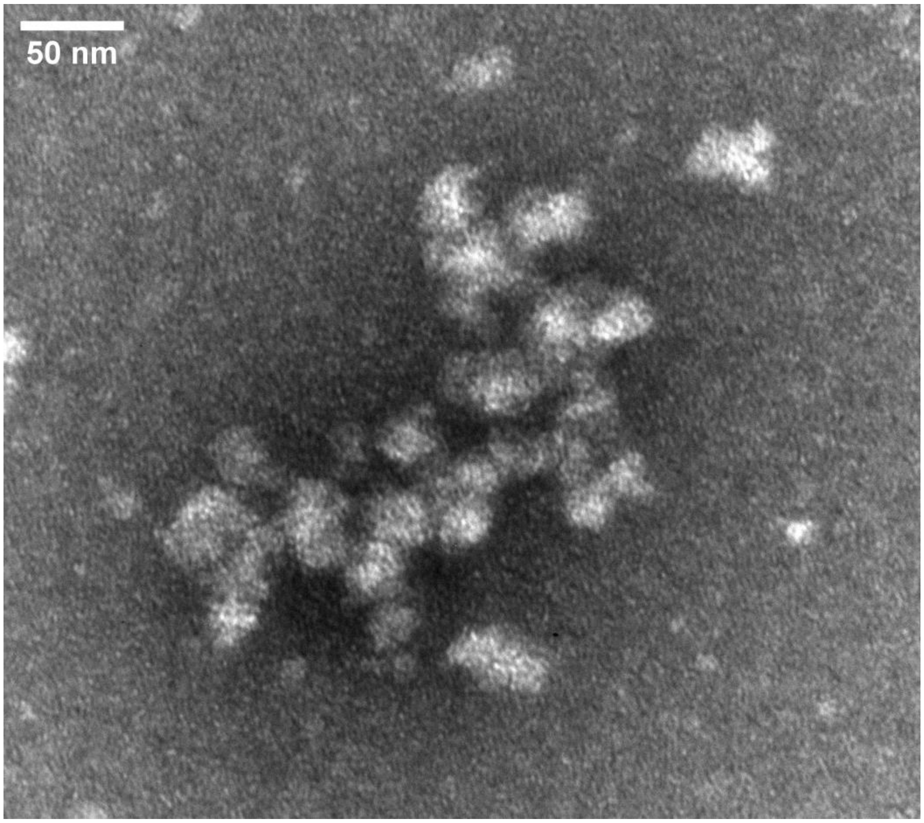

Figure S12: TEM micrograph of nanogels N50 taken at 80 KeV.

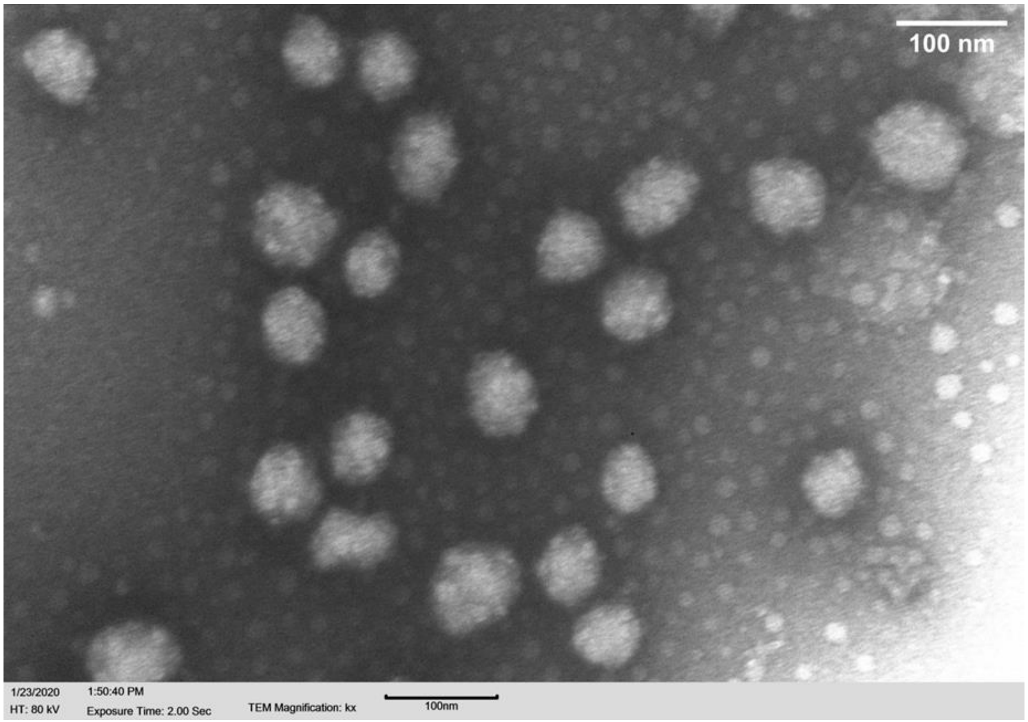

Figure S13: TEM-micrograph of nanogels N48 taken at 80 KeV.

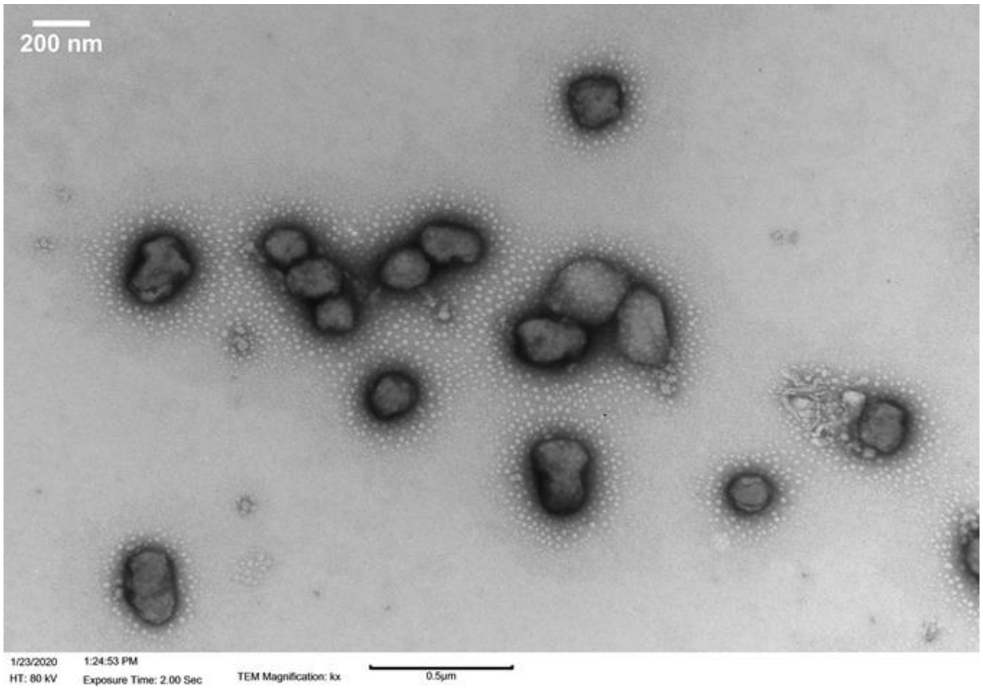

Figure S14: TEM micrograph of nanogels N45 taken at 80 KeV.

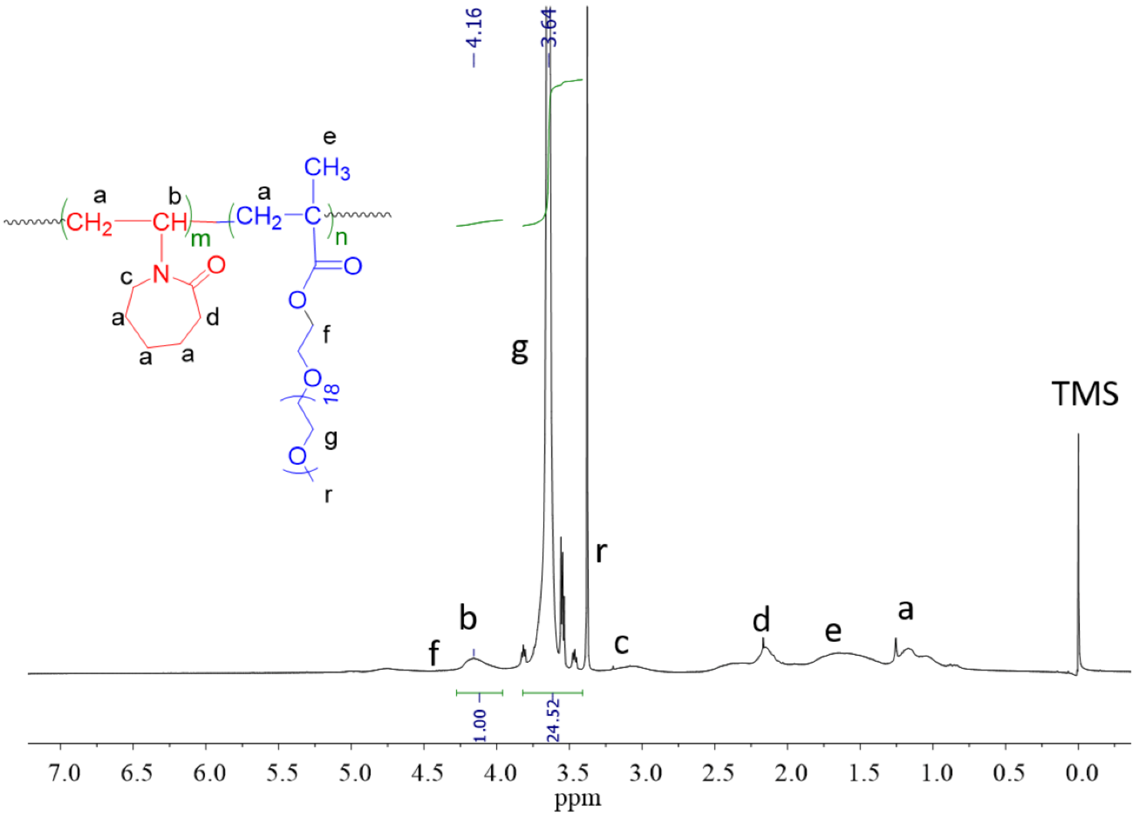

Figure S15. ^{1}H-NMR spectrum of nanogel N32.

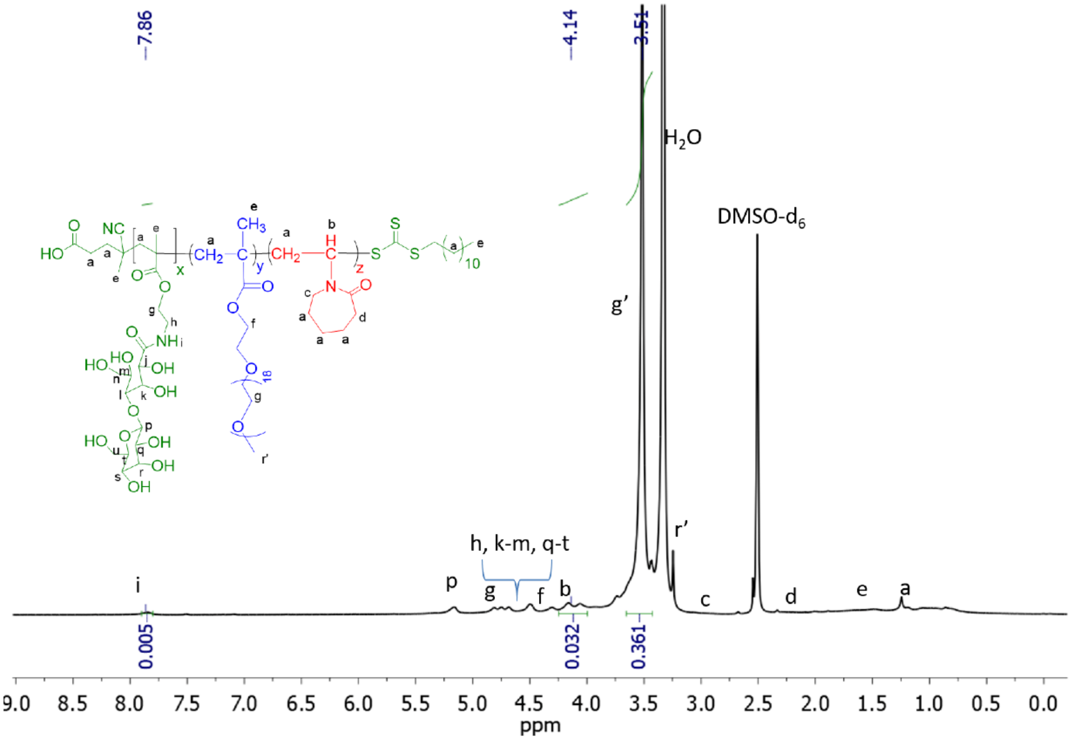

Figure S16. ^{1}H-NMR spectrum of nanogel N44.

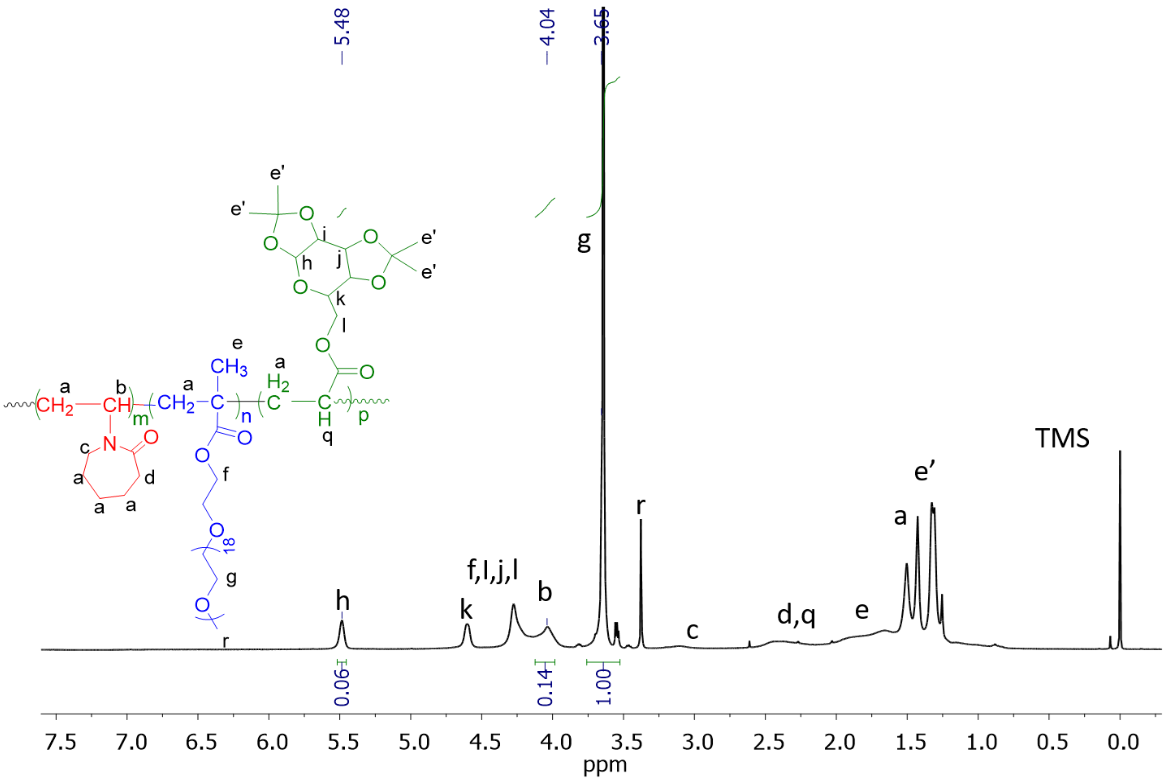

Figure S17. ^{1}H-NMR spectrum of nanogel N46.

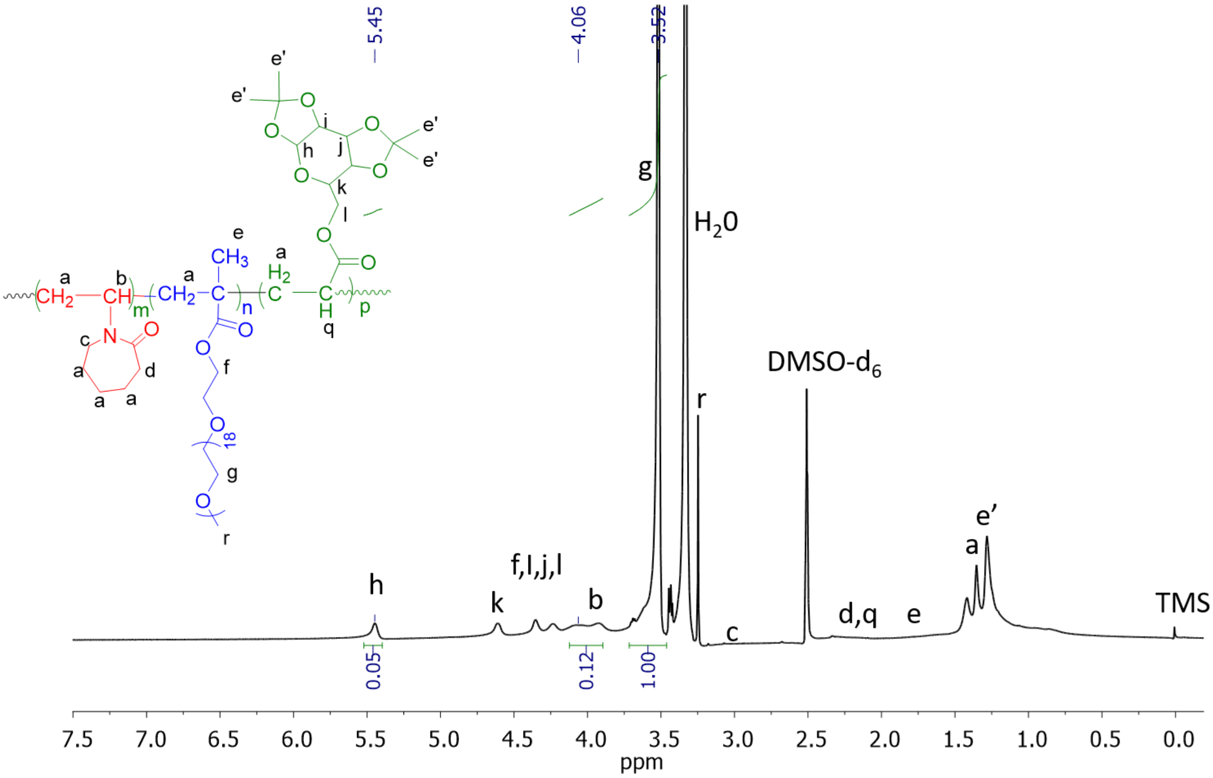

Figure S18. ^{1}H-NMR spectrum of nanogel N45.

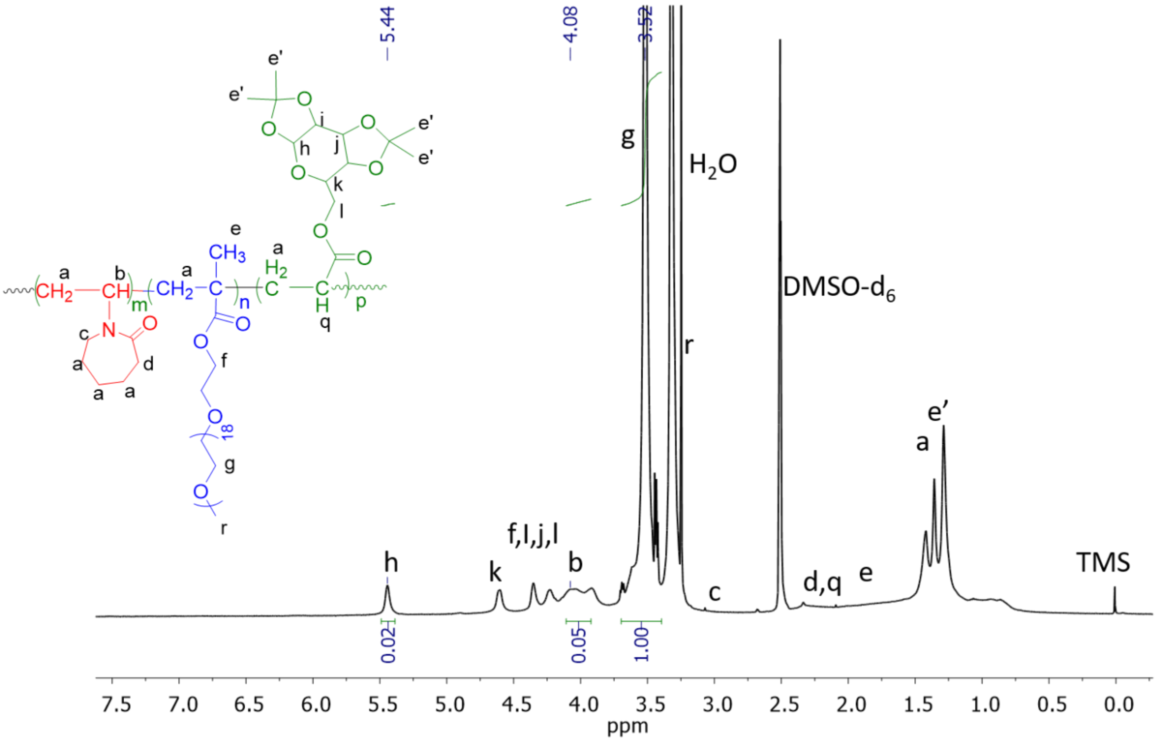

Figure S19. ^{1}H-NMR spectrum of nanogel N48.

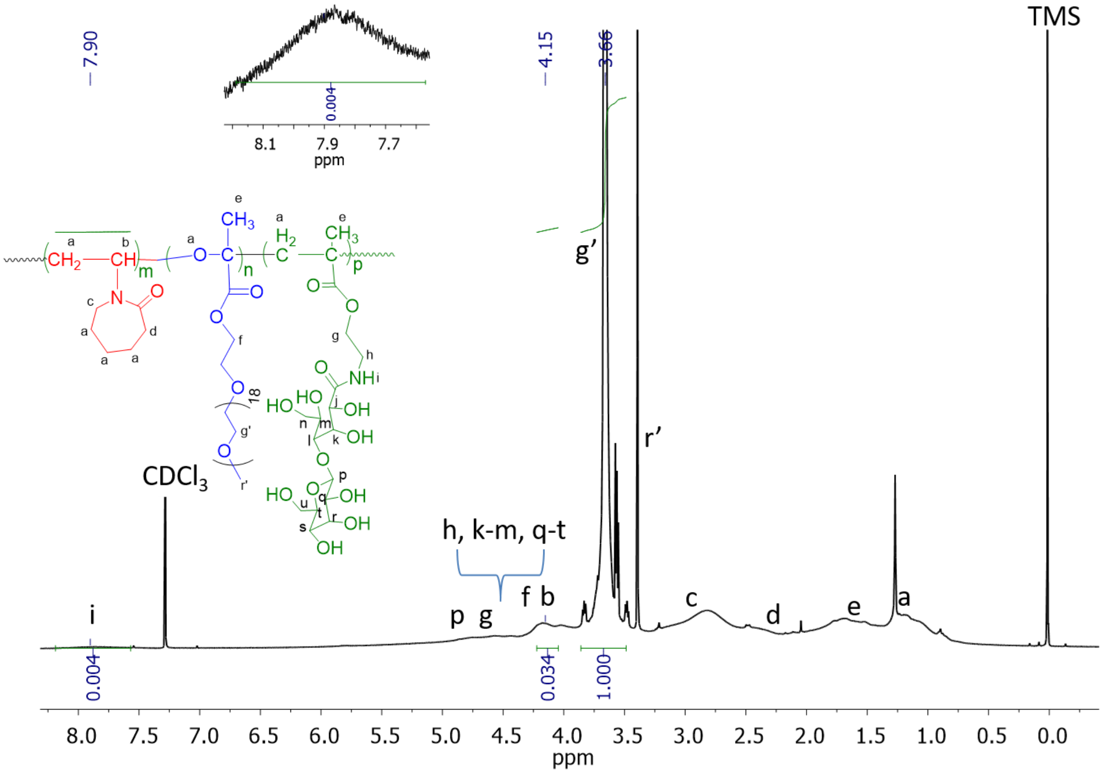

Figure S20. ^{1}H-NMR spectrum of nanogel N50.

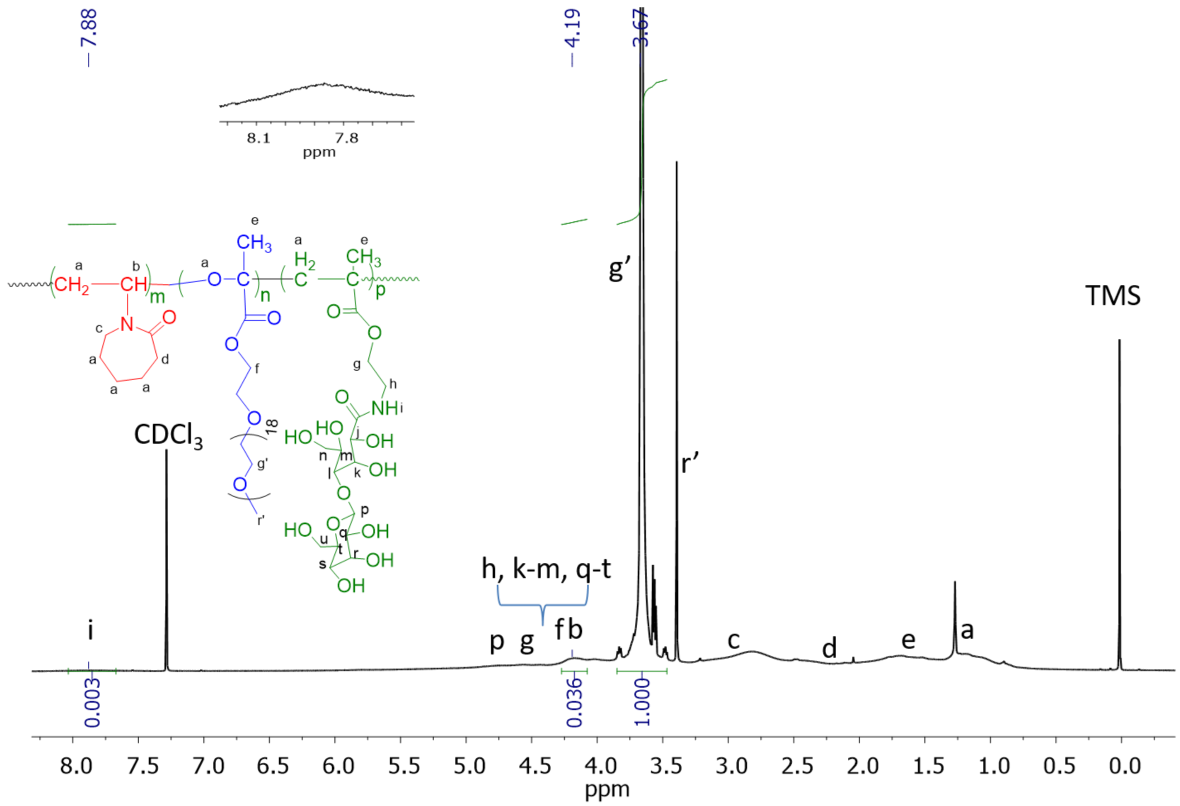

Figure S21. ¹H-NMR spectrum of nanogel N51.

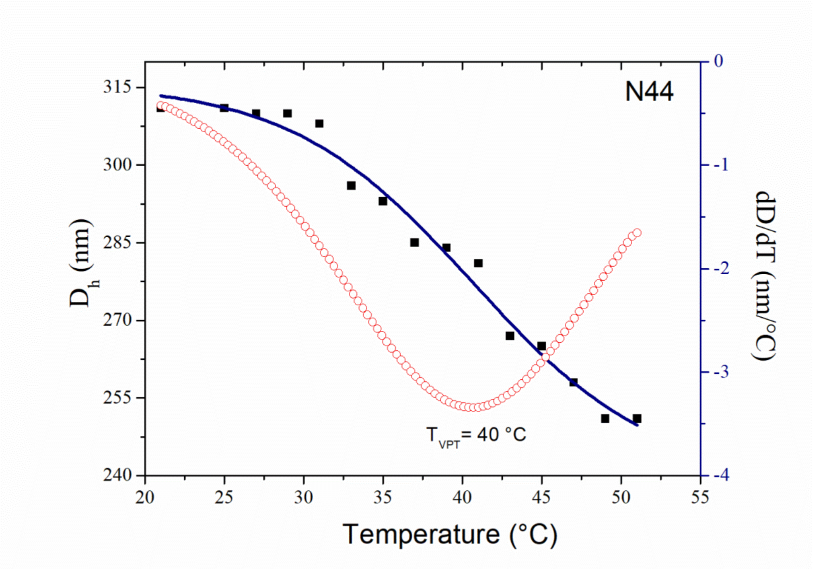

Figure S22. D_h of nanogel N44 as function of temperature obtained by DLS.

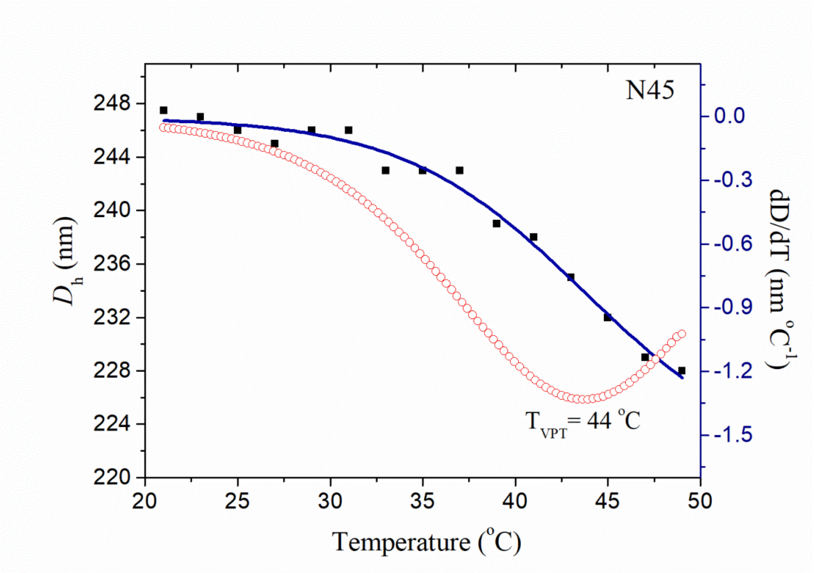

Figure S23. D_h of nanogel N45 as function of temperature obtained by DLS.

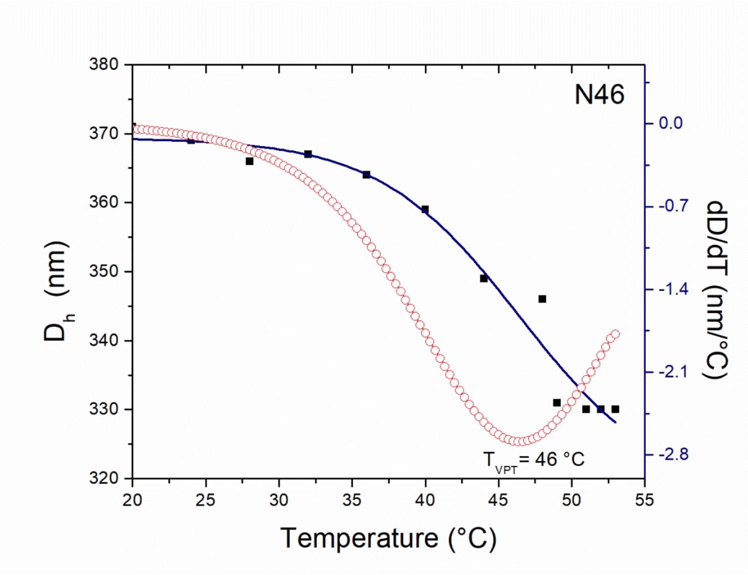

Figure S24. D_h of nanogel N46 as function of temperature obtained by DLS.

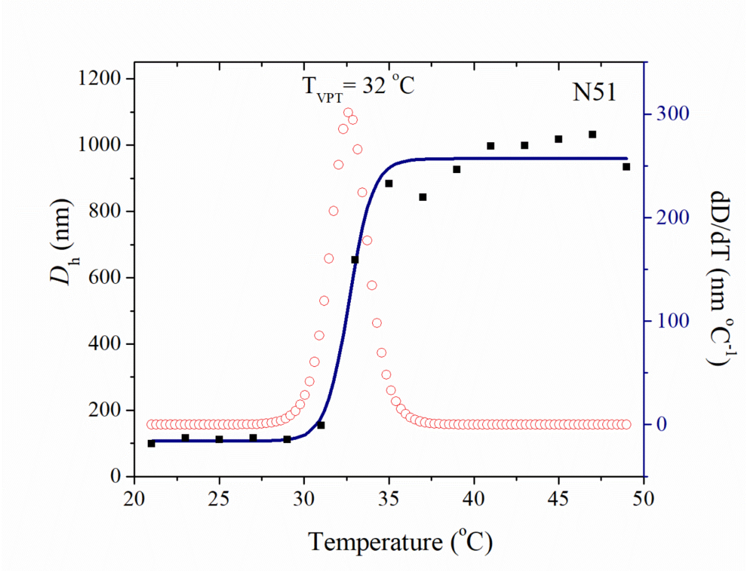

Figure S25. D_h of nanogel N51 as function of temperature obtained by DLS.
